# Synergistic interactions among growing stressors increase risk to an Arctic ecosystem

**DOI:** 10.1038/s41467-020-19899-z

**Published:** 2020-12-07

**Authors:** K. R. Arrigo, Gert L. van Dijken, M. A. Cameron, J. van der Grient, L. M. Wedding, L. Hazen, J. Leape, G. Leonard, A. Merkl, F. Micheli, M. M. Mills, S. Monismith, N. T. Ouellette, A. Zivian, M. Levi, R. M. Bailey

**Affiliations:** 1 School of Earth, Energy & Environmental Sciences, Stanford University, Stanford, CA USA; 2 Stanford Center for Ocean Solutions, Stanford University, Stanford, CA USA; 3 School of Geography and the Environment, University of Oxford, Oxford, UK; 4 The Ocean Conservancy, Santa Cruz, CA USA; 5 Hopkins Marine Station, Stanford University, Pacific Grove, CA USA; 6 Department of Civil and Environmental Engineering, Stanford University, Stanford, CA USA; 7 Center for Advanced Study in the Behavioral Sciences, Stanford University, Stanford, CA USA

**Keywords:** Systems biology, Environmental sciences, Ocean sciences

## Abstract

Oceans provide critical ecosystem services, but are subject to a growing number of external pressures, including overfishing, pollution, habitat destruction, and climate change. Current models typically treat stressors on species and ecosystems independently, though in reality, stressors often interact in ways that are not well understood. Here, we use a network interaction model (OSIRIS) to explicitly study stressor interactions in the Chukchi Sea (Arctic Ocean) due to its extensive climate-driven loss of sea ice and accelerated growth of other stressors, including shipping and oil exploration. The model includes numerous trophic levels ranging from phytoplankton to polar bears. We find that climate-related stressors have a larger impact on animal populations than do acute stressors like increased shipping and subsistence harvesting. In particular, organisms with a strong temperature-growth rate relationship show the greatest changes in biomass as interaction strength increased, but also exhibit the greatest variability. Neglecting interactions between stressors vastly underestimates the risk of population crashes. Our results indicate that models must account for stressor interactions to enable responsible management and decision-making.

## Abstract (web summary)

Multiple co-occurring stressors may affect food webs in ways that are not predictable by studying individual stressors. Here the authors apply a network interaction model to a marine food web in the Arctic, finding that nonlinear interactions between stressors can more than double the risk of population collapse compared to simpler simulations.

## Introduction

The multiple environmental stressors associated with global climate change and human activities (e.g., shipping, fishing, pollution, etc.) are dramatically impacting ocean systems, specifically the functions and ecological services they provide^1,2^. Ecosystem modeling is a key tool for understanding and predicting major shifts in ecological communities. Traditionally, ecosystem models have incorporated impacts from two or more stressors by summing their individual effects on a species or ecosystem, multiplying two stressors together without isolating the interaction effect, or applying (potentially arbitrarily) weighting factors^3–7^. However, ample evidence demonstrates that multiple stressors frequently interact in a non-additive manner^8–10^. Synergistic interactions, where the combined impact of stressors is greater than the sum of their individual effects^11,12^, are of particular concern because of their greater potential for harm. Indeed, a meta-analysis of multiple stressor studies found that simple additive interactions occur in only 26% of studies, with synergistic (amplified effects) and antagonistic (dampened effects) interactions occurring in 36 and 38% of studies, respectively^8^. However, direct experimental quantification of stressor effects on organisms and ecosystems is limited to individual stressors and a few pairwise interactions^8,13–15^. There is an urgent need to analyze how suites of multiple co-occurring stressors impact entire food webs in order to anticipate possible tipping points and focus management interventions on the most deleterious stressor combinations^16–19^.

Existing models provide relatively fine-scale predictions of how individual stressors affect whole ecosystems^15^. However, synergistic interactions between stressors can yield non-linear impacts^11^ that when unaccounted for may underestimate risk to organisms. Here we use a novel modeling framework—the Ocean System Interactions, Risks, Instabilities, and Synergies (OSIRIS) framework^20^, [Supplementary Methods]—to explore the possible outcomes of stressor interactions on populations and ecosystems in the face of global change. When applied to an interconnected ecosystem network, this modeling framework can account for the effect that each possible stressor pairing has on each type of organism with a unique interaction term.

We focus on the Arctic Ocean because it is changing rapidly^21,22^ as a result of warming, causing ecological upheaval^21^ and opening a “new ocean”^23,24^ for commercial and industrial development. Unprecedented Arctic warming is increasing marine primary productivity^25,26^ while endangering iconic species and disrupting global ocean circulation^27,28^. Arctic Ocean ecosystems are simultaneously impacted by rapid changes in temperature, freshwater content, nutrient concentrations, pH, and sea ice cover that strongly affect ice-dependent species^29–38^. Sea ice melt also exposes the Arctic Ocean to the expansion of development and marine transportation, introducing a new suite of anthropogenic stressors to the region that will add to and potentially accelerate escalating climate stressors. In particular, increased ship traffic facilitated by the loss of sea ice could significantly impact marine life by increasing the incidence of ship strikes, underwater noise, water pollution, and entanglement in marine debris^39–41^.

**Fig. 1:**
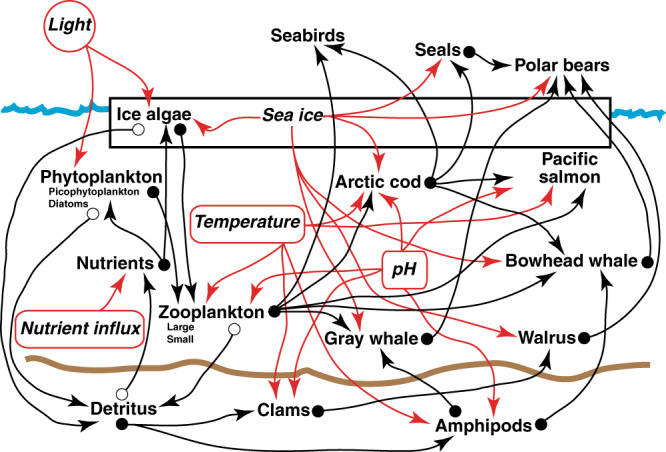
Schematic of the modeled Arctic network model. Dynamic state variables or nodes (non-italicized labels) respond to changes in daily-defined stressors (red arrows), changes in adjacent node values (black arrows), and the node-carrying capacity at each time step. Closed circles represent bidirectional connections; open circles represent unidirectional connections where the change in the target-node state does not affect the source-node state. Pairwise stressor interactions are possible among any two forcings that target the same node and are uniquely defined for each node and stressor pairing. Node names represent the dominant species in a functional group (i.e., Arctic cod are the dominant small fish in the Chukchi Sea, although other small fish are also present). If no species is dominant within a functional group, then the name used is more generic (e.g., Clams). Shipping noise and strike stressors, as well as subsistence harvest of fish and marine mammals (Table 1), are not shown.

Within the Arctic marine environment, we use the Chukchi Sea ecosystem as a case study and examine its behavior over a 20-year period (2020–2040) to address the near-term changes that are most relevant to management and policy interventions in the region. The Chukchi Sea, situated off the coast of northwest Alaska, is one of the most productive ecosystems in the world and is characterized by a relatively simple food web (Fig. 1) compared to many other marine ecosystems^42–44^. Thus it is an ideal system for understanding the impacts of non-linear interactions among different environmental stressors on key marine species.

Here we show that chronic stressors (e.g., sea ice loss, decreased pH, warming seas) were more harmful to ecosystems than acute stressors (e.g., shipping noise and strikes, subsistence harvest). Moreover, neglecting synergistic interactions between multiple stressors can result in a dramatic underestimate of the probability of population collapse within this fragile environment.

## Results

### Baseline simulation

The baseline simulation used our best estimate of all model parameters and the strength of all interactions between model variables gleaned from the literature. During this 20-year simulation, mean annual sea ice concentration declined by 14%, the length of the open water season increased from 135 to 165 days, maximum annual sea surface temperature (SST) increased from 3 to 4 °C, and ocean pH dropped from 8.1 to 8.0 (Supplementary Fig. 1). Nutrient flux was assumed to be proportional to the volume flux through Bering Strait^45^. Shipping noise and strikes were assumed to increase approximately sixfold over the course of the 20-year simulation due to increased shipping activity through the Bering Strait^46^.

Due primarily to the decrease in sea ice cover over the central Chukchi Sea, ice algal biomass in the region was predicted to decline 20% by the end of the 20-year baseline simulation (Supplementary Fig. 2). However, this decline in sea ice algal biomass was more than compensated for by the 41% increase in picophytoplankton and the 33% increase in planktonic diatoms that resulted from the increased open water area over that same 20-year period (Supplementary Fig. 2).

The responses of higher trophic level organisms in the baseline simulations were more complex due to their greater number of connections to other components of the model. Increased SST and greater phytoplankton biomass resulted in a relatively minor increase in small zooplankton biomass (1.2%) by the end of the 20-year simulation but a larger increase in large zooplankton (16%), due primarily to the consumption of small zooplankton by large zooplankton (Supplementary Fig. 2). Similarly, animals living in the sediments also benefitted from higher temperatures and a greater phytoplankton food supply, as evidenced by the 1.3% increase in clams and 8.4% increase in amphipods over the 20-year simulation (Supplementary Fig. 2).

It might be expected that Arctic cod, which feed on large zooplankton, would increase in response to their greater food supply, but this was not observed. Instead, Arctic cod populations declined by 21% due primarily to their dependence on a shrinking sea ice cover and to a lesser extent in response to increases in SST and ocean acidification. Because Pacific salmon were assumed to feed on both a declining population of Arctic cod and large zooplankton, their populations declined as well but to a lesser degree (8.9%) due to the increased availability of their large zooplankton prey and their higher growth rate at increased temperatures. Seabirds also feed on large zooplankton and Arctic cod, so their populations fell by a similar percentage as Pacific salmon (10%).

The five marine mammal species included in our model all declined by similar amounts after 20 years in our baseline simulation, albeit for different reasons. The 9.1% seal decline was driven mostly by the loss of sea ice but also by the reduction in Arctic cod abundance. Walrus populations declined to a similar degree (10.1%) due to sea ice loss, despite the small increase in the clams on which they feed. Polar bear populations dropped 10.2% due primarily to the decline in their seal prey but also to the additional noise from increased shipping activity. Unlike the other marine mammals, the declines in bowhead whales (9.3%) and gray whales (10.6%) were due primarily to acute stressors such as increased noise and ship strikes and, in the case of bowhead whales, continued subsistence harvesting.

### Effects of interaction strength on biomass

**Fig. 2:**
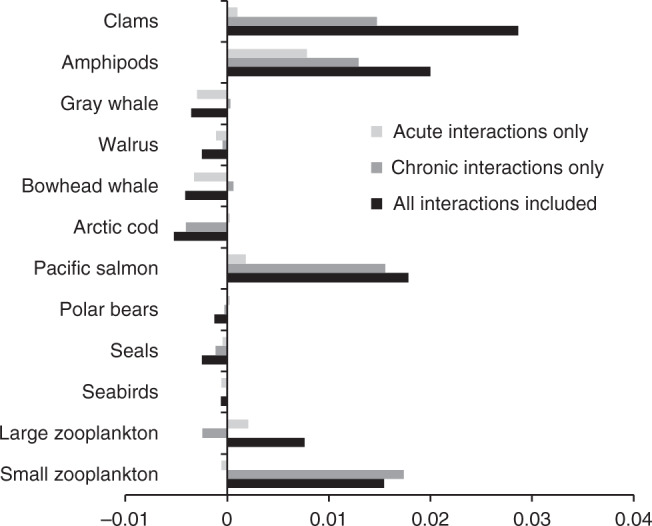
The sensitivity of the biomass of each node to interaction strength. This was calculated as the slope of the relationship between the mean final biomass of each node and changes in interaction strength, which varied from antagonistic to synergistic. Example data from which the slopes were calculated are shown in Fig. S3. A separate set of simulations was run with only acute stressor interactions included, only chronic stressor interactions included, and all stressor interactions included. Positive numbers indicate that biomass increased with increasing interaction strength.

Because the previous simulation was run at a baseline interaction level for all stressor combinations (Table 1 and Supplementary Table 4), we wanted to better understand how changes in the strength of these stressor interactions would impact population sizes in the Chukchi Sea (no stressor interactions were assumed for ice algae or phytoplankton). At the end of these 20-year simulations, the change in mean biomass of all organisms as a function of interaction strength fell into two distinct categories (black bars in Fig. 2)—organisms that declined slightly as interaction strength increased and became more synergistic (seabirds, seals, polar bears, Arctic cod, bowhead whales, gray whales, and walrus) and those that increased substantially as interaction strength increased (small and large zooplankton, amphipods, clams, and Pacific salmon). Both of these categories included organisms with different numbers (1–4) and combination of stressor interactions (Table 1) that drove their population changes, so the sensitivity of population size to changes in interaction strength did not depend on the number of interactions considered. In general, all organisms whose biomass increased with increasing interaction strength were those that responded positively to the increase in SST during the 20-year simulations (all the invertebrate groups plus Pacific salmon). The lone exception was Arctic cod, which was also assumed to grow faster at higher SST (but only up to 2 °C), but whose population declined with increasing interaction strength. The reason for this decline is that Arctic cod are even more sensitive to the amount of open water area than they are to SST, and therefore the decline in sea ice concentration over the 20-year simulation drove their populations downward. The decline in sea ice cover, in combination with subsistence harvesting pressure, was also responsible for the drop in the populations of the other large organisms included in the model (seals, polar bears, bowhead whales, gray whales, and walrus) as interactions became more synergistic.

**Table 1:** Stressor interactions used in the simulations.

Variable	Interacting stressors
Small and large zooplankton	SST × pH
	SST × inflow
	pH × inflow
Amphipods and clams	SST × pH
Pacific salmon	SST × pH
	SST × subsistence harvest
Arctic cod	SST × pH
	SST × open water duration
	Shipping noise × open water duration
	Subsistence harvest × open water duration
Seabirds	Shipping noise × shipping strikes
Seals, walrus, bowhead and gray whales	Shipping noise × shipping strikes
	Shipping noise × open water duration
	Subsistence harvest × open water duration
Polar bears	Shipping noise × open water duration
	Subsistence harvest × open water duration

There are no interactions assumed for ice algae and phytoplankton.

We also ran two other sets of simulations, one that only included interactions between chronic stressors and one that only included interactions between acute stressors. This allowed us to determine which type of stressor is likely to be more important in controlling the marine ecosystem of the Chukchi Sea. For most of the organisms whose biomass increased with interaction strength (small zooplankton, Pacific salmon, amphipods, and clams), the results for simulations that included only chronic stressor interactions (dark gray bar in Fig. 2) were very similar to those that included all interactions (black bars in Fig. 2). This indicates that those organisms were markedly less sensitive to acute stressors (light gray bars in Fig. 2) than to chronic stressors at increasing interaction strength. Furthermore, higher biomass at higher interaction strength was always tied to SST—warmer waters led to higher growth rates despite decreases in pH or changes in any other stressor. Organisms whose biomass dropped with increasing strength of stressor interactions, a subset that included walrus and bowhead and gray whales, responded more strongly to acute stressors, especially ship strikes and ship noise, than to chronic stressors (light gray bars in Fig. 2). In contrast, seals and Arctic cod, which are less susceptible to ship strikes, responded most strongly to the loss of sea ice, a chronic stressor.

### Effects of interaction strength on biomass variability

**Fig. 3:**
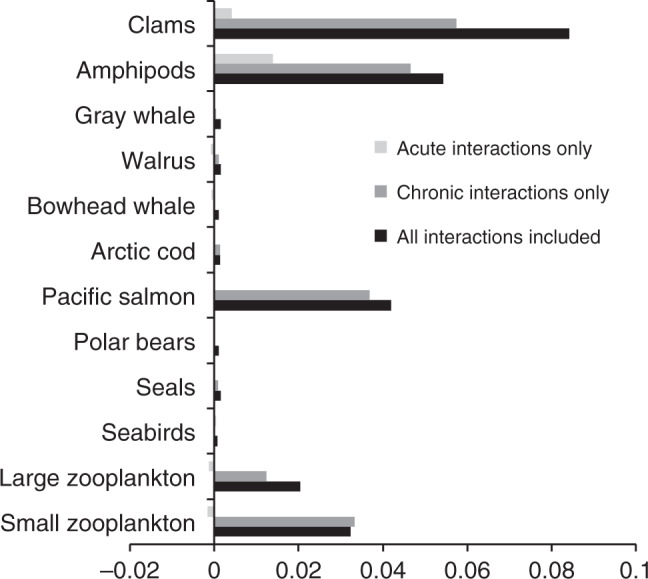
The sensitivity of the variability of each node to interaction strength. This was calculated as the slope of the relationship between the variability in final biomass of each node and changes in interaction strength, which varied from antagonistic to synergistic. Example data from which the slopes were calculated are shown in Supplementary Fig. 4. A separate set of simulations was run with only acute stressor interactions included, only chronic stressor interactions included, and all stressor interactions included. Positive numbers indicate that biomass variability increased with increasing interaction strength.

For all organisms, the standard deviation of the mean biomass for the 200 simulations at each stressor interaction level increased with increasing interaction strength when all interactions were included (black bars in Fig. 3), although for many, the increase in variability was small. Those organisms showing the greatest variability in biomass with increased interaction strength also exhibited the greatest increase in biomass at increasing interaction strength (Fig. 2), suggesting that, with increasing interaction strengths, effects were overall positive but also less predictable. When all interactions were included, the standard deviation in the biomass of small and large zooplankton, Pacific salmon, amphipods, and clams increased by approximately 3-, 2-, 5.5-, 5-, and 3-fold, respectively, as interaction strengths increased from their lower to their upper bounds. The increase in biomass variability with interaction strength virtually disappeared for all organisms when only acute stressors were included (light gray bars in Fig. 3), indicating that interactions between chronic stressors (dark gray bars in Fig. 3) were more important in controlling the variability in organism biomass than acute stressors.

### Probability of population collapse versus interaction strength

**Fig. 4:**
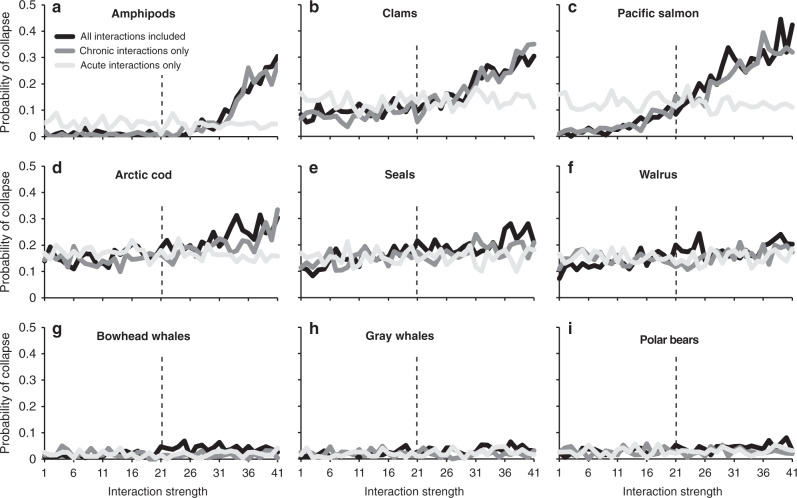
The probability of population collapse plotted as a function of interaction strength. Included are (**a**) amphipods, (**b**) clams, (**c**) Pacific salmon, (**d**) Arctic cod, (**e**), seals, (**f**) walrus, (**g**) Bowhead whales, (**h**) Gray whales, and (**i**) polar bears. A separate set of simulations was run with only acute stressor interactions included, only chronic stressor interactions included, and all stressor interactions included. The dashed line represents the baseline interaction strength.

At the end of the 20-year simulations, a number of Arctic marine organisms exhibited a >10% risk of population collapse when both chronic and acute stressor interactions were included (black lines in Fig. 4). For clams, Arctic cod, seals, and walrus, the probability of population collapse was consistently near or above 0.1 and increased with interaction strength. For amphipods and Pacific salmon, the probability of population collapse was low at low interaction strengths (when stressor interactions were antagonistic) but increased dramatically to 0.3–0.4 at greater interaction strengths where interactions between stressors are most synergistic. This represents a 40- and 3.7-fold increase in the risk of collapse over the baseline simulations, respectively, for these two groups. The risk to organisms that were less sensitive to interaction strength was calculated to increase from 1.1- to 2.6-fold. Notably, the sensitivity of population collapse to interaction strength disappeared when only acute stressors were included (light gray lines in Fig. 4), demonstrating that the interactions between chronic stressors were driving the increased risk of collapse with increasing interaction strength (dark gray lines in Fig. 4). While acute stressors were also associated with a substantial risk of population collapse for some organisms, this risk did not increase as interaction strength increased.

Some organisms had a higher risk of collapse at lower interaction strengths when only acute stressors were included (chronic stressors were switched off), such as Pacific salmon, amphipods, and clams (light gray lines in Fig. 4). This is because the lower bounds for the interaction strength of chronic stressors for these organisms were negative (antagonistic), meaning that when chronic stressors (particularly pH and SST) were included, they partially negated each other at these low interaction strengths, reducing the risk of collapse. As interaction strength increased above baseline values, the interactions between chronic stressors became synergistic and the risk of collapse increased dramatically (dark gray lines in Fig. 4).

## Discussion

Although it is known that the cumulative impact of escalating stressors, and particularly their synergistic interactions, poses major threats to the stability of ecosystems, to our knowledge no model to date has explored a range of interaction types and strengths among multiple stressors. Here we explore a range of interaction types and strengths among numerous stressors in the Chukchi Sea sector of the Arctic Ocean. Our results indicate that synergistic interactions among key stressors can more than double (in some cases much more) the risk of population collapse compared to that predicted in simulations without non-linear interactions. In the coming decades, the sheer magnitude and changing nature of anticipated threats present an unprecedented potential to alter the Arctic Ocean environment permanently. While precautionary ocean management is warranted in the face of this uncertainty, it is currently unclear how extensive precautionary measures must be to protect ocean services^14^.

Our results suggest that organisms in the Chukchi Sea vary markedly in their sensitivity to multiple interacting stressors. Some studies have shown that the sensitivity of state variables to external stressors may be related to the overall connectedness of that state variable to other components of the model, with more connections leading to greater stability^47–49^. This can mask the destabilizing role played by external stressors and lead to erroneous conclusions about ecosystem resiliency. In the OSIRIS model of the Chukchi Sea, the total number of connections for each organism ranged from 4 (for clams and amphipods) to 11 (for zooplankton) (Fig. 1). Plotting the probability of population collapse against the number of connections for each organism showed that there was no relationship between the two (Supplementary Fig. 5a). This remained true when the number of organism–organism interactions (Supplementary Fig. 5b) and organism-forcing variable interactions (Supplementary Fig. 5c) were assessed separately. Similarly, the number of non-linear interactions assumed for each organism ranged from 1 (for clams and amphipods) to 4 (for Arctic cod). Again, there was no relationship between the number of non-linear interactions for an organism and the risk of population collapse (Supplementary Fig. 5d), even when chronic (Supplementary Fig. 5e) and acute stressors (Supplementary Fig. 5f) were considered separately. Thus we are confident that our results are a consequence of the biological responses included in the OSIRIS model rather than an artifact of model connectedness.

Modeling of stressor interactions in an Arctic ecosystem revealed some intriguing patterns in how food webs may respond, and how different types of stressors may drive these patterns. Large organisms at higher trophic levels were generally negatively impacted by increasing stressor interaction strength, while lower trophic levels tended to increase in biomass (Fig. 2). This may be due to large, long-lived, and mobile organisms integrating the effects of many stressors and their interactions over their different life stages and habitats. However, the variability in the response to stressors by large organisms is small (Fig. 3), reducing their probability of population collapse (Fig. 4). In contrast, small planktonic or benthic organisms may generally benefit from warmer SST, increased primary productivity associated with ice loss, and their combination. Chronic stressors, such as SST, pH, and ice loss, also tended to dominate effects over acute stressors like subsistence harvesting and ship noise and strikes, again suggesting that the sustained exposure over the life cycle of organisms increased the likelihood of interactive effects on the resulting biomass and its variability. While the mechanisms underlying population responses to interacting stressors should be further investigated, these simulations offer some initial insights.

Three insights emerge from the modeling results described above. First, synergistic interactions amplify adverse stressor effects on ocean ecosystems. Second, the impact of synergy is predicted to increase with the magnitude of stressors, which are anticipated to grow with expanded human activities. Third, synergies will make population and ecosystem responses more unpredictable. Quantifying the range of synergistic impacts is necessary to understand the limitations of single stressor management and support holistic ecosystem-based management in the face of accelerating climate change. Our results indicate that an increase in synergy strength amplifies internal model variability and thus leads to a broader range in possible outcomes, even when stressors themselves do not change. It is reasonable to assume that synergistic interactions among some species or ecosystem stressors exist^8–10^, and thus neglecting them provides a false sense of certainty as to the range of possible model outcomes. These implications make investigating interactions among stressors crucial for our understanding of ecosystem risk and thus for designing policies to lower the social, economic, and biological consequences of environmental change.

While the trends described in these model scenarios are direct predictions of ecosystem trajectories given the best available data, what is more important is that they demonstrate the potential role of uncertainties in a region undergoing extreme change. This uncertainty comes in different guises. Given increased stressor levels and synergistic interactions, for example, the model indicates that there is more variance in biomass loss. Additionally, there is uncertainty in the estimates of organismal responses to stressors as well as how much stressor levels and their interactions will increase. For example, gauging the effect of increased shipping on bowhead whales over the next 20 years is difficult; there are limited data on how ship strikes may affect this species, although empirical evidence from other species may inform the response^50,51^. Likewise, bowhead whale responses to increases in noise levels are even more difficult to estimate^52–57^. The impacts of noise pollution are widely recognized and show a variety of effects, from masking communication signals to potentially lethal consequences; this variability is difficult to capture in a single parameter^56–61^. Thus our study suggests that any amount of assumed impact from a single stressor will likely be affected by more complex interactions with other stressors, some of which are poorly understood. These effects may be larger than is currently recognized, suggesting that we may underestimate future impacts of multiple simultaneous stressors^11^. If even weak synergistic effects prove to be common, an additional level of caution amid the substantial changes in the Arctic will likely be warranted. The results presented here allow us to explore the influence of the nonlinear effects on potential biomass loss as well as uncertainty and point to areas where more focus is required. Additionally, they highlight the potential benefit that may be gained by minimizing increases in different stressors.

Quantification and mapping of cumulative impacts of multiple stressors on marine ecosystems have shown that vast areas of the oceans are already highly impacted^62,63^ and that these impacts are rapidly escalating^64^. However, to our knowledge previous analyses do not account for nonlinear interactions among stressors and potential synergies. Here we show that synergistic stressor interactions have the potential to substantially increase the risk of population collapse and ecosystem shifts. This study is an early step in understanding the relative response of species and ecosystems to stressor interactions in a region where climate change is causing rapid change and highlights the danger of underestimating risk when non-linear interactions are neglected. While not all interactions will prove to be significant in a modeling study, those that arise from large stressor effects or act upon key ecosystem species warrant more research. For these, the interactive effects could outweigh the direct effects of other modeled stressors. In particular, our result that chronic stressor interactions tend to dominate suggests that, under continued warming, ocean acidification, and ice loss, the impacts of synergies are predicted to also escalate. Our findings spotlight important research needs and management implications. Future research should aim to identify possible levers for mitigating impacts, by, for example, identifying the stressors that more frequently and significantly interact synergistically with others^65^. As such, research that increases our understanding and capacity to anticipate tipping points should be prioritized^16,18,19,66,67^.

## Methods

The OSIRIS model^20^ represents ecological systems as a network of interconnected nodes. Nodes are defined as representations of groups with shared characteristics, which may be biotic or abiotic. Biotic nodes can represent age cohorts, species, functional groups, or different spatial populations, and abiotic nodes can represent chemical (e.g., nutrient elements) or physical (e.g., light) variables. The treatment of nodes is flexible to adapt to the ecosystem in question at an appropriate resolution. Biotic nodes represent biomass and are linked up in the network based on trophic relationships. The model is assumed to be at equilibrium. For additional technical details, see Supplementary methods.

We examined the impact of stressor interactions on amplifying or dampening uncertainties within components of the modeled Chukchi Sea ecosystem by exploring the range of model outcomes as stressor interactions vary from antagonistic to synergistic. For each modeled species or species group (nodes, Fig. 1), we determined the appropriate range of parameter values (Supplementary Table 1) to describe its growth and death in response to environmental forcing variables (Supplementary Fig. 1) and interactions with other organisms. We also determined the baseline and upper and lower bounds for the strength of the interactions (negative values denote antagonism and positive values denote synergism) between different model forcing variables for each organism (Supplementary Table 4). Forcing variables that can act interactively include both chronic stressors that are largely climate related, such as sea ice loss, SST, pH, and change in inflow from the Bering Sea, and acute stressors from direct human interactions, like harvesting, ship noise, and ship strikes. All stressor interactions (Table 1) were simultaneously and proportionally scaled from their lower-bound values to their upper-bound values, becoming more synergistic as they increase. We divided the range of the strength of interactions (between the lower and upper bounds) for each organism into 41 discrete segments and ran 200 simulations at each interaction segment, with each simulation using a different randomly sampled set of parameter values (using Latin hypercube sampling) from the range of values determined for each organism that control growth, death, and interactions with other organisms.

Results for the last year of the 20-year simulations are presented as the mean and standard deviation in biomass calculated for each organism over the 200 simulations as a function of interaction strength. The standard deviation, signifying the spread in model outcomes, arises from the 200 different internal model parameter combinations. We also calculated the probability of population collapse after 20 years for each organism (defined as the fraction of the 200 simulations at each synergy strength where the biomass of a population fell below 10% of its initial value) as a function of interaction strength. Three sets of simulations were run: one set with both chronic and acute stressor interactions included, one set with only chronic stressor interactions, and one set with only acute stressor interactions. This allowed us to determine the relative importance of the best-estimate model parameters for chronic and acute stressors and their interactions on populations of many of the most abundant and ecologically relevant species in the Chukchi Sea, as well as investigating how changes in the strength of the interactive stressors impact population biomass change.

The model was forced using environmental data for the next 20 years (2020–2040) that have been extrapolated from observations made at the central Chukchi Sea over the past few decades. These observations include seasonal changes in incident light, SST, sea ice concentration, open water duration, ocean pH, nutrient flux through Bering Strait, shipping noise, probability of ship strikes, and the level of subsistence harvesting (Supplementary Fig. 1). The same forcing profiles were used in all OSIRIS runs. Validation of model results can be found in the Model validation section (S5) of the Supplementary information.

### Reporting summary

Further information on research design is available in the Nature Research Reporting Summary linked to this article.

## Supplementary information

Supplementary Information

Reporting Summary

## Data Availability

Model results presented in this paper and forcing data used to drive the OSIRIS model are available at the permanent Stanford Digital Depository (https://purl.stanford.edu/js210vj5991). The source data underlying Figs. 2–4 and Supplementary Figs. 1–5 are provided as a Source data file, also located at the Stanford Digital Repository.

## Source data

Source Data

## Code availability

Code for the Arctic version of the OSIRIS model is available at the permanent Stanford Digital Depository (https://purl.stanford.edu/js210vj5991). The available files include README_OSIRIS.docx, configuration spreadsheet, example configuration files, and Matlab code.
