# Supplementary material for: Synergistic interactions among growing stressors increase risk to an Arctic ecosystem
Source: Nat Commun. 2020 Dec 7;11:6255. doi: 10.1038/s41467-020-19899-z (PMC7721797; doi:10.1038/s41467-020-19899-z)
Supplement: Supplementary file 1 — Supplementary Information [file 41467_2020_19899_MOESM1_ESM.pdf]

Supplementary Information

Supplementary Methods

S1. OSIRIS Model Description

OSIRIS is a coupled ordinary differential equation model and the primary outputs are node time series [1]. Nodes are represented by a single state variable. In the case of biotic nodes, these represent biomass. Biotic node states (except plankton nodes) are normalized to unity under baseline forcing conditions (at equilibrium, this means that node states are at 1, or 100%). Phytoplankton and zooplankton nodes are normalized by the average first-year biomass values, which was done because these nodes seasonally vary strongly. Nutrients and detritus are treated similarly as the plankton nodes. The state of each node is locally stable around an equilibrium value (carrying capacity or physical equilibrium). Nodes are externally forced with different stressors. These stressors affect the node equilibrium value (e.g., carrying capacity). Through trophic connections, effects are carried through the network.

One fundamental assumption of OSIRIS is that there is a system-level equilibrium condition, where all nodes are at their equilibrium. At the system-level equilibrium (assuming favorable conditions), there is no net influence between interacting nodes. When nodes are forced out of the equilibrium state, they have net effects on other connected nodes. As such, the model is described as a network of 'net influences' on node states. OSIRIS does not include all elements of an ecosystem as there is no explicit energy or mass balance to maintain. This assumption of an intrinsic stable state was empirically documented during stable climate regimes of the Holocene and is the starting point for much theoretical work [2, 3].

Nodes tend to return to their equilibrium state (Supplementary Figure 6). The logistic model describes this dynamic for biotic nodes (asymmetrical potential with S-shaped growth recovery), while abiotic nodes have a recovery rate that is proportional to the deviation from equilibrium (symmetrical potential, with exponential response). Some biotic nodes are also ascribed a non-stable equilibrium point, which represents the Allee effect; i.e., below the non-state equilibrium

point, a population may experience positive feedback that limits recovery or accelerates decline and extinction.

The state of node j (n_j) and its dynamics are determined by three components: (i) the rate of relaxation towards local equilibrium (R_j); (ii) the direct influence of nodes on other nodes, and (iii) the influence of external forcings on node states. The effect of (ii) and (iii) are combined into one parameter characterising interactions (I_j):

$$f(n) = \frac{dn_j}{dt} = R_j + I_j \quad \text{(Eq. 1)}$$

where R_j represents the recovery of a biotic node without Allee effect (Eq. 2a), with Allee effect (Eq. 2b), or the recovery of an abiotic node (Eq. 2c):

$$R_j = \begin{cases} n_j r_{0_j}\left(1 - \frac{n_j}{k_j(\Phi)}\right) & \text{(Eq. 2a)} \\ n_j r_{0_j}\left(1 - \frac{n_j}{k_j(\Phi)}\right)\left(\frac{n_j}{A_j} - 1\right) & \text{(Eq. 2b)} \\ r_{0_j}\left(k_j(\Phi) - n_j\right) & \text{(Eq. 2c)} \end{cases}$$

where r_{0_j} is the intrinsic recovery/growth rate, Φ represents the combined effect of multiple forcing: $\Phi_j = f_\varphi\left(\varphi_{1,j}, \varphi_{2,j} \ldots \varphi_{N_F,j}\right)$, k_j represents the equilibrium state and is a function of Φ, and A_j represents the Allee effect.

Interactions between one node (source node) and another node (target node) that affect node state are defined as

$$I_j = \Gamma_j n_j. \quad \text{(Eq. 3)}$$

Γ_j incorporates all effects that directly affect n_j, the inter-node influences.

n_j changes over time, which is calculated as:

$$n_j(t) = \int_0^t f(n)\, dt \quad \text{(Eq. 4)}$$

The integration is solved numerically using 4th-order Runge-Kutta integration.

The equilibrium state of node j (k_j) is influenced by forcing F_m according to the functional form of $\varphi_{m,j}$. The choice of function for $\varphi_{m,j}$ depends on data (if available) or on theory. In the Chukchi Sea model, relevant functions were low-order polynomial and Gaussian functions:

$$\varphi_{m,j}(F_m) = \begin{cases} \sum_{q=0}^{n_q} a_q F_m{}^q & \text{(Eq. 5a)} \\ a\left[\left(exp\left(-(F_m-b)^2/(2c^2)\right)\right)\right]+d & \text{(Eq. 5b)} \end{cases}$$

where a, b, c, d are fitting parameters (we set d=0 for all nodes in all of our runs).

When two or more external forcings simultaneously affect a node state, their values ($\varphi_{m,j}$) are combined at each time increment (0.05 days) as either additive or multiplicative effects. The results yield Φ_j (as required in Eq. 2). Eq. 6 illustrates the combination of effects to calculate an overall effect (y):

$$y = a_1x_1 + a_2x_2 + a_3x_3 + b_1x_1x_2 + b_2x_1x_3 + b_3x_2x_3. \qquad \text{(Eq. 6)}$$

Coefficients a_i (additive) and b_i (multiplicative) indicate the nature of the combined effect, where $b_i > 1$ represents synergistic effects, $b_i < 1$ represents antagonistic effects, and $b_i = 0$ represents no multiplicative effect. Only pairwise combinations are incorporated in the model, as opposed to triple combinations, because empirically-based parameterisation is available for only pairwise condition. Including more than two factors increases the uncertainty in effects.

S2. OSIRIS Arctic Parameterisation

S2.1. Node-node interactions

Internal interactions represent the effect a node has on another node and vice versa when one or both are out of equilibrium. When both nodes are in equilibrium, there is no net effect. Interactions are based on trophic relationships. When prey or predator nodes are increasing or declining in biomass, away from their equilibrium, then their connected predators or prey nodes will experience positive or negative effects, depending on the interactions. For example, prey populations will benefit when predator populations decline. This results in an increase in prey

biomass, which in turn may benefit their predators. Specific seasonal behavior, especially for the phytoplankton nodes, means that nodes are often out of equilibrium. In fact, the rise in phytoplankton in response to the return of light results in increases in zooplankton, in agreement with natural dynamics in the Chukchi Sea.

The trophic relationships are calculated based on the percentage dietary composition of each predator node. The term predator is used here in the broadest sense as fish eating zooplankton, zooplankton grazing on phytoplankton, and phytoplankton taking up nutrients. All prey nodes (in the broadest sense) that contribute to the diet of a predator are identified. The percentage that each prey node contributes to a predator's diet, as estimated from the literature, was scaled so that the sum of the dietary composition percentages for each prey adds up to 1, and all represent positive effects. This value is then weighted by the daily intrinsic growth rate of the predator (Supplemental Table 1) in order to get the predator influence on prey (Supplemental Table 2). To determine the negative effects (predator influence on prey), the sum of all positive effects a prey node gives was first calculated. For each interaction that a particular prey node has with a predator, this positive influence is divided by the total positive effects a prey node has across all predators, and this is multiplied by -1 to create a negative effect. This method means that all negative effects of each predator node sum up to -1. Similar to the value of predator effect on prey, these values are then weighted by the daily intrinsic growth rate of the prey to get the prey effect on the predator (Supplemental Table 2).

Last, growth rates for each species are determined from literature and are either empirically derived or theoretically established (Supplemental Table 2). These rates represent the maximum growth rates a node can reach. If these values were represented as annual rates, they were divided by 365 days to obtain a daily growth rate. These values are multiplied by the positive and negative interactions for each specific node to obtain the internal interaction values for each node.

S2.2. Node responses to specific forcing

Several forcings were included in the model to represent characteristic Arctic seasonal conditions, as well as long-term changes. Node-specific responses to different forcing were either empirically informed or based on expert opinion. Forcings include sea surface temperature, pH, open water duration, light, shipping noise, ship strikes, and harvest expected over the next 20 years (harvest was assumed to remain constant over that period). The model time step was 0.05 days. Some forcings represent chronic climate stressors (pH, temperature, and open water duration) while others represent acute anthropogenic stressors (ship strikes, shipping noise, and harvest). Light availability changes as a function of ice fraction, the time series of which is specified in the model. It controls the light availability for ice algal production (and implicitly influences their available habitat) and light availability for pelagic phytoplankton production.

To parameterize the model, it was first determined how each node would be impacted by each stressor (Supplemental Table 3). Second, parameterizations of each node for a specific stressor set were established following the equations described above. Parameterization methods for each stressor are outlined below.

Temperature: Empirical evidence for species expected to be influenced by temperature changes were obtained from the literature (see Supplemental Table 3 for values and references). Based on the studies provided, all nodes affected by temperature, except the phytoplankton nodes, were ascribed to follow a Gaussian distribution response [4]. The phytoplankton nodes were assumed to respond linearly based on empirical evidence (Supplemental Table 3). Temperature changes over the 20-year simulations were based on historical trends obtained from Optimum Interpolation Sea Surface Temperature (OISST v2, https://www.ncdc.noaa.gov/oisst), with an annual change of +0.017°C.

pH: Based on the previously published empirical studies, the slope between pH treatments and organismal response was calculated. A straight line is expected following previous work on pH responses [5] and this slope was used to describe a node's response to pH changes (see

Supplemental Table 3 for values and references). Expected change in pH over 20 years followed projections from Mathis et al. [6], with an annual change of -0.005 pH units.

Open water duration: Open water duration (OWD), or the number of ice-free days per year, is expected to influence many organisms but in different ways. For example, while it is expected that sea ice loss will be detrimental for polar bear populations (e.g., loss of hunting and denning places), an understanding of the relationship between ice-free days and polar bear biomass change is unknown. Here, we assume a small negative linear relationship for the specific nodes that are likely to be sensitive to increases in OWD (Supplemental Table 3). The projected changes in OWD were based on Barnhart et al. [7]. See Table S3 for more specific details on ice melt rates and ranges chosen.

Light: Light availability was determined for ice algae and pelagic phytoplankton. Responses to changes in light were based on the amount of photosynthetically active radiation (PAR) calculated using a clear sky model corrected for cloud cover and were further dependent on ice fraction. Ice fraction changed throughout the year (as opposed to OWD, which was used as an annual measure) and followed historical satellite-based observations of freezing/melting rates for the Chukchi Sea. The daily change in sea ice is important to include as spring light levels drive the productivity of the system during the rest of the year.

Ship strikes: Ship strikes have been calculated based on data from bowhead whales, but are assumed to be equal for the other whale species in the model, the grey whale. It is assumed that only whale species may be lethally hit in this model. The effect of ship strikes is calculated as follows. The proportion of the population affected by ship strikes, and assumed dead (thus, a loss in biomass) is dependent on the probability of collision and the probability that the hit is lethal, which in turn is influenced by ship speed, the amount of shipping, and whale population size. Based on surveys from legally harvested bowhead whales, scar data have shown that at least 2% of the whale population has been hit by a ship [8,9]. These non-lethal ship strikes are assumed to be an underestimation of the whale population that experiences a strike. The 2% is used as a conserved estimate for the percent of the whale population that is hit by a ship. Lethality of the

collision is dependent on ship speed. While the relationship between ship speed and lethality has not been studied for Arctic whales, it has been for North Atlantic right whales [10], a closely-related species. Using that model, we calculate the effect of ship strikes on whales. The average ship speed in the Arctic is taken as 14 knots [11], resulting in a proportion of lethal strikes of 0.79. According to the IUCN, the Bering-Chukchi-Beaufort population of bowhead whales has recovered well from whaling in the previous century. It is currently close to or at carrying capacity and is set to 1 (100%). The effect of ship strikes on population loss is controlled by the shipping magnitude; current shipping magnitude is set to 1, and the magnitude changes linearly with projected increases in shipping (Supplemental Table 3). Shipping is expected to increase 6-fold over the next 20 years [12]. See S4.1 for more specific details on ship strikes and how it was treated.

Shipping noise: Shipping noise is an acute anthropogenic impact that acts on marine organisms, mostly mammals. However it is not necessarily lethal. It can cause a suite of responses, including behavioural changes and increased energy costs (which are not mutually exclusive) [8]. Behavioural changes include abandoning feeding areas, changing migration routes, increasing diving responses, changing call frequency (whales), and leaving resting places (pinnipeds). Increased energetic costs may result from increased travel times, diving, or other avoidance responses. Shipping noise likely exacts a cost to organisms in the form of increased stress [9,13]. Combined, these factors likely affect population biomass through a reduction in reproductive success; the increase in stress and energetic costs can even result in mortality. While data on the effects of shipping noise are limited for bowhead whales, there are data for North Atlantic right whales. The two species are very similar in morphology and life history [8]. However, unlike the bowheads, which live in a relatively low anthropogenically impacted environment, the North Atlantic right whales often live in industrialized regions where they are exposed regularly to the impacts of shipping. It is suggested that this exposure affects their recovery potential and that the species is currently still recovering from whaling. Because of their small population, however, individual North Atlantic right whales have been well studied,

and effects of shipping and other stressors have been well documented. The ship noise forcing strength (assumed to be linear) is taken to be equal or less to the effect of ship strikes (Table S3). This stressor, like ship strikes, is assumed to increase 5-fold over the 20-year period. See S4.2 for more specific details on shipping noise and how it was treated.

Harvest: The harvest forcing refers to the take of marine animals such as mammals and salmon, and it is based on the potential biological removal, although is not assumed to be solely legal harvest (as often the actual harvest is lower than that advised). The forcing is based on the latest U.S. marine mammal stock assessments [14], which calculate annual potential biological removal as the product between the minimum population estimate, half the (theoretical) population growth rate, and a theoretical recovery factor. These rates are converted to an average daily removal rate (Supplemental Table 3). This forcing is kept constant during all simulations.

S2.3. Node responses to stressor interactions

Multiple simultaneous stressors can interact, resulting in antagonistic or synergistic effects. Such interactions are being increasingly documented [15], although they are still poorly understood. For example, pairwise interactions do not necessarily correlate in their effect with more than two stressors [16]. However, there is very limited empirical evidence documenting the effects of more than two stressors. Hence OSIRIS has been built to incorporate only pairwise interactions and thus reduce the number of unknown model parameters.

Interactions between stressors are determined per stressor per node (Supplemental Table 4). Where possible, these interactions are empirically informed via literature. When data were not available (for example, between the shipping stressors), expert opinion influenced the effect magnitude. Empirically informed interactions were calculated by determining the effect of the two single variables alone and the added increase or decrease from having both variables together.

The results of several meta-analyses investigating the effect of pairwise stressors on organisms highlight the widespread presence of interactive effects of multiple simultaneous

stressors [16-21]. Many single-species, single-stressor experiments have highlighted the negative impacts stressors may have on particular organisms. For example, acidification leads to decreases in calcification, growth, development, abundance, and survival in many marine organisms, although the strength of the responses varies between phyla [19]. However, the effects of multiple simultaneous stressors are not necessarily additive, but instead are interactive (i.e., multiplicative) as evidence from meta-analyses on pairwise stressors indicate [16, 17, 20]. Roughly a third of pairwise stressors are synergistic, while the proportion of antagonistic effects can vary between 38-43% [16, 17, 20]. However, these values may change depending on what life stage is investigated: Przeslawski et al. [21] found that in early marine life stages, 65% of multiple simultaneous stressors had synergistic effects, while 17% of all multiple simultaneous stressors were antagonistic.

Whether there is a predictable average effect of multiple simultaneous stressors, and whether this is synergistic or not is still up for debate. Crain et al. [16] found that on average, the interactive effect in their meta-analysis was synergistic, while Darling & Côte [17] contested this and found no such evidence in their meta-analysis. The differences in results can be explained by the way additive effects are modeled to determine the presence, direction, and magnitude of an interaction effect [21]. Other results may be more informative; there is likely a difference between trophic levels in terms of the average effect of multiple simultaneous stressors. Both Crain et al. [16] and Harvey et al. [18] found that the average interactive effect of multiple simultaneous stressors in phototrophs was antagonistic, while it was synergistic for heterotrophs. As mentioned above, there is also likely a difference in the sensitivity to multiple simultaneous stressors between different life stages [18, 21].

Furthermore, the responses to multiple simultaneous stressors vary between phyla [18, 21]. Like single-stressor experiments, however, pairwise stressors are a simplification of the real world. The addition of a third stressor can increase the proportion of synergistic interactive effects [16]. Inter-species interactions may change a specific species' response to a particular set of stressors [19].

The presence, magnitude, and direction of an interactive effect of multiple simultaneous stressors are dependent on the biological variable being measured. Several meta-analyses investigating pairwise stressors showed some consistency in terms of the type of interactive effect and measured biological variables. Harvey et al. [18] showed that multiple stressors acted synergistically on calcification, photosynthesis, reproduction, and survival while growth did not. Kroeker et al. [19] report that survival, growth, and development had pairwise stressors acting synergistically on them while calcification did not. Przeslawski et al. [21] report that calcifying organisms are more sensitive to interactive effects than non-calcifying organisms measured for the same biological variables. As the magnitude of the interactive effect can vary depending on the choice of biological variable, it is important to consider this choice. A change in mortality under multiple simultaneous stressors is an ultimate integrative measure, as all sub-lethal changes will be incorporated in this biomass loss. However, other variables, such as calcification, do not necessarily lead to a decrease in population but is expected to affect calcifying organisms negatively.

All factors described above contribute to the uncertainty around the estimate of interactive values of multiple simultaneous stressors, be they for specific species or functional groups and life stages. Because the true interactive value may be uncertain, if there is such an estimate, it is important to incorporate the variability in the estimate. In order to incorporate this variation, we created different ranges around the interactive estimate for different pairwise stressor combinations per species or functional group included in our model. These ranges varied in terms of what biological variable was used to inform it and studies included to inform the estimate. We created a literature range based on experiments using Arctic species and a wider range of biological variables and studies from polar regions and in few occasions, other geographical areas (Supplemental Table 4), especially for those Arctic groups that had limited information available. The reason why we included estimates from other geographical regions is that the calculation of the interactive value (described below) results in a unit less interaction

coefficient, and the results we obtained did not vary much from the interaction coefficients obtained from Arctic studies.

In order to calculate the interaction coefficients for pairwise stressors for different biological variables, a study needed to include a control treatment, treatments of both stressors alone, and a treatment of both stressors combined. The average effect of a particular biological variable was determined, and the difference between the two single treatments and the control treatment was used to calculate the additive estimate. The additive effect was determined by the direction of the effect of two single stressor estimates. If both stressors yielded a higher estimate than the control, the additive effect was calculated by adding the absolute differences between the control and each single treatment to control, while if the two single stressor estimates yielded lower estimates than the control, the additive effect was calculated by subtracting the absolute differences between the control and the two single treatments from control. When the two single stressor estimates were in the opposite direction, this was incorporated in the calculation of the additive effect. The additive effect should be equal to the estimate of the combined treatment if there was no interactive effect present between the two treatments.

The interaction coefficient was calculated as follows:

$$X = \frac{(\mu_{AB} - \mu_{A+B})}{(\mu_A \times \mu_B - 1)} \quad \text{(Eq. 7)}$$

where X represents the interaction coefficient, μ_{AB} represents the estimate of the combined treatment, μ_{A+B} represents the estimate of the additive effects, μ_A represents the estimate of treatment A, and μ_B represents the estimate of treatment B.

Several studies reported on the effects of more than one biological variable, and in some cases multiple treatment levels in one treatment were used. For example, Kunz et al. [22] investigated the effects on survival, oxygen consumption, growth rate, and relative feed conversion in Arctic cod under 0, 3, 6, and 10°C in combination with two pH levels (current pH 8.05 and future pH 7.6), which were all included to inform the literature range and highlight the uncertainty around the interaction coefficient.

S3. Impacts of Sea Ice Thinning and Loss

Sea ice in the Arctic is changing both in extent and thickness as a result of global warming [23]. Sea ice has an important function in the Arctic; it functions as a habitat (e.g. ice algae), refuge (e.g., juvenile Arctic cod), resting place (e.g. seals and walruses), hunting platform (e.g. polar bears), reproductive site (e.g. seals and walruses; also in combination with snow, especially for ringed seals which form the majority of polar bear diet), and it influences the soundscape in the water by dampening the noise. Therefore, the loss of ice will have a significant impact on the biological community [24-29]. Ice extent throughout the years has declined as the ice pack melts earlier and freezes later in the year in response to warming conditions. The open water duration in the Chukchi Sea has been increasing with 9-12 days per decade since the 1980s, and possibly higher [30], while across the Arctic, the average increase in OWD is roughly five days per decade [31]. Douglas [32] predicted that the median increase in OWD would be roughly 1.5 days for the next 20 years, although there was a large spread around the median, ranging from no change to roughly 7-8 days of an increase in OWD per year. Serreze et al. [33] estimated a similar increase in OWD of 1.5 days per year for the Chukchi Sea as well as for the last three decades. Onarheim et al. [34] determined that, based on satellite data, the Chukchi Sea could be ice-free in the summer by 2050, resulting in a 1.55-day increase in open water duration per year. While the projections of future ice loss are uncertain, it is likely that these estimates are not extreme. Climate models tend to provide lower estimates, yet these models often underestimate observed trends [34].

Across the Arctic Ocean, multiyear sea ice has been declining since the 1980s, resulting in a loss of 30% in areal extent of this ice type [35]. The oldest age classes of ice have been replaced by 2- and 3-year old ice, which now makes up the majority of multiyear sea ice [36]. In various parts of the Arctic, the sea ice thickness has been reduced by 0.5-1.5 m [37]. Young sea ice melts earlier in the season than does multiyear ice and it also allows for greater light penetration [38-40]. Light availability influences the start of seasonal marine primary production, which will

occur earlier in the year as a result of earlier and increased light availability, although it is influenced as well by the increased ice loss earlier in the year [37, 40]. The Chukchi Sea, for example, has already seen an increase in marine primary productivity by 42% [41, 42]. The change in phenology and primary productivity may, in turn, affect the zooplankton that grazes on the phytoplankton, as well as having further knock-on effects on organisms that depend on the zooplankton for survival [43].

S4. Impacts of Shipping

The Arctic Ocean currently is and has been relatively inaccessible to ships for large parts of the year. The presence of sea ice, therefore, has played a protective role for many larger organisms, like whales, which can be harmed by ships through physical impact as well as noise pollution. As a result of the loss of ice and the resulting increase in open water duration, shipping activity will increase in volume, type, and duration in the future, resulting in increased risks of shipping-related impacts for many organisms [44-47]. However, there is a lack of data and understanding of how an increase in shipping activity may impact the animals in this region [45]. Ship strikes, for example, can be non-lethal or lethal. It can be difficult to estimate how many whales may die from ship strikes. Scarring data obtained from legally harvested bowhead whales show the low occurrence of propeller-related injuries, but it is possible that individuals that are severely injured are lost to the population, and thereby were missed in the estimate [48]. A study investigating the impacts of propeller injuries in North Atlantic right whales showed that 50% of these injuries were fatal [49]. Not all ship strikes will result in a physical mark. Ship strikes may result in blunt trauma, which does not leave a scar, but can result in death [50-52].

Other whale species that live in industrial shipping areas can be informative about some of the shipping-related impacts and potential future for Arctic whales. Noise pollution is however, still a difficult and understudied aspect of shipping in terms of quantitative damage. While there are many qualitative impacts described, such as displacement, increased stress, and even some noise pollution has been linked to mortality, it remains difficult to quantity the full range of

impacts that result in abundance or biomass loss. Below, the stressors are investigated in more detail to illustrate how the impacts used in the OSIRIS model were derived.

S4.1. Impacts of Shipping – Strikes

Vessel strikes have been identified as a major threat to whale species worldwide [53]. Vessel strikes can result in injury and death; the result is dependent in part on ship speed [54]. Currently, the Bering Sea-Chukchi Sea-Beaufort bowhead whale population is not threatened by ship strikes. This population has suffered from whaling practices in the last century but has recovered to or close to preindustrial levels [44, 55]. This is in stark contrast to a closely related species, the North Atlantic right whale, which is currently one of the most threatened whale species, with around 400 individuals left. Ship strikes have been identified as a major factor limiting the recovery of this species [52, 53, 56]. North Atlantic right whales have a higher chance of being killed by a ship strike than any other large whale species [50]. Changes in the food distribution of North Atlantic right whales, as a result of climate change, are expected to put this species at an even higher risk of ship strikes [57].

Speed restrictions as well as shipping restrictions to reduce co-occurrence have been identified as the most successful strategies to minimize the loss of endemic whales in anticipation of the increase in Arctic vessel traffic [58, 59]. North Atlantic right whales can be instructive as to what could happen to bowhead whale populations in the future as the two species are very similar in reproduction, migration strategies, feeding, and other characteristics such as swimming speed, morphology, and longevity [44]. It is expected that bowhead whales will, therefore, respond to ships in a similar way as the North Atlantic right whales. North Atlantic right whales have been extensively studied, and the information and models generated for this species are used here as a best-estimate to understand how bowhead whale populations may respond to increased shipping in the Arctic. Vanderlaan & Taggart [54] estimated the probability that a ship strike would be lethal based on the ship’s speed. Their logistic regression estimated that the largest change in the probability that a ship strike would be lethal was between

speeds of 8.6 and 15 knots, where the chance goes up from 0.21 to 0.79. Speeds above 15 knots asymptotically approach 1, or a 100% chance that a ship strike is lethal. Speeds below 11.8 knots would result in a 50:50 chance that a hit would be lethal. Increases in speed, likely to happen in the Arctic Ocean as the hazardous conditions that ice create are disappearing, will have different impacts on the odds that a strike would be lethal. An increase in 1 knot increases the odds by 1.5-fold, while an increase in 2 knots would increase the odds 2.3-fold, and an increase in 5 knots increases the odds 7.9-fold [54].

While certain characteristics of a whale, like age, size, or swimming behavior, and a ship, like type, size or angle of the collision, also influence the lethality of the strike, although these data are often not available. However, the mass of the whale and the speed of the ship are likely the most important factors determining the impact of the strike, and while other factors play a role (such as the duration of the collision and the energy dissipation), it is likely that vessel speed is one of the most reasonable predictors for ship strike lethality [54].

The logistic regression of Vanderlaan & Taggart [54] was sparse in data for low-speed collisions. Conn et al. [126] investigated the relationship between ship speed and the probability a ship strike is lethal for North Atlantic right whales along the east coast of the U.S. They found a similar relationship as did Vanderlaan & Taggart [54], although the difference was mainly that Conn et al. [60] estimated higher chances for lethal strikes for speeds lower than 15 knots than did Vanderlaan & Taggart [54]. For example, the estimated probability that a hit would be lethal for ship speeds of 8-9 knots was 0.5 in Conn et al. (compared to the 0.21 of Vanderlaan & Taggart [54]). Speeds higher than 13-14 knots showed that the two logistic regressions were more likely similar, with the Vanderlaan & Taggart [54] regression estimate falling within the 95% confidence interval of Conn et al. [60]. Conn et al. [60] included two new observations of lethal strikes at low speed, resulting in the more extreme estimate compared to Vanderlaan & Taggart [54]. It is, therefore, possible that even at low speeds, hits can result in high whale losses. However, data of lethal strikes at low speeds remain limited, and drawing major conclusions is difficult.

The logistic regression estimates based on the North Atlantic right whales are used here to estimate ship-strike effects on bowhead whales. The effect of ship strikes is determined by incorporating the population of whales that is hit by a ship, the chance a hit is lethal, which is dependent on the vessel speed, and the amount of shipping present in the Arctic. This is formally represented as follows:

$$B_i = H_i L S \qquad \text{(Eq. 8)}$$

where B_i represents the relative biomass loss for species i as a result of ship strikes, H_i represents the proportion of the population of species i that is hit by ships, L represents the chance that the hit is lethal, which in itself is dependent on ship speed and is looked up in the logistic regression estimates, and S represents the relative amount of shipping in the Arctic, where 1 represents current shipping levels.

The population of bowhead whales that is hit by a ship is difficult to determine, but based on scar data from legally harvested whales, about 2% of the whales show scars of ship-related injuries. This estimate is taken as a conservative estimate for the whole population for several reasons. As described above, not all ship strikes will leave marks (blunt trauma does not necessarily leave a scar, resulting in a lower estimate for ship strikes). In addition, severely injured whales may be lost before the harvest and thus not accounted for (resulting in a lower estimate for ship strikes). Finally, observational scar data may not reliably be attributed to a ship-related event and thus were not included in the estimate, which again can result in underestimating the occurrence of ship strikes in the bowhead whale population [9, 44]. The chance that a hit is lethal is determined by using the regression described above and established by Vanderlaan & Taggart [54]. Ship speed in the Arctic is influenced by the presence and thickness of ice, as well as vessel type. Several ships operate in the Arctic Ocean, including bulk carriers, containers, icebreakers, special purpose, passenger, tankers, tug and barge, and general cargo ships. The number of ships of each type varies from 11 to 321 for special purpose to container ships, and their average speed varies from 10 to 17 knots for tug and barge and

icebreaker ships [11]. The model does not differentiate between ship types, and thus average ship speed for all types was used to determine the chance that a hit would be lethal. The average ship speed was 14 knots [11], resulting in a probability of 0.79 that a ship strike is lethal, which is similar to the estimate of Conn et al. [60].

BOEM et al. [61] used ship speeds of 4.5 (towing gear) to 16.5 knots (transiting) in their analyses of the impact of shipping noise on Arctic organisms. Pine et al. [62] investigated in their study what they assumed was the baseline speed of 25 knots in the Arctic and what effects this speed would have as noise impact on marine mammals and Arctic cod compared to a 10-knot speed reduction. The noise impacts will be further discussed in the next section, but it is worth noting that shipping speeds in the Arctic can become higher, getting closer to a possible 100% chance that any hit would be lethal. The suggested 10-knot speed reduction would still mean a high chance that a ship strike is lethal. McWhinnie et al. [53] report in their review that current vessel speed in the Arctic Ocean is lower than other open ocean areas as a result of ice hazards. Thus, the Pine et al. [62] data should be interpreted with caution, although the call for the benefits of speed reductions are valuable. Ships will be able to travel faster when they are not limited by sea ice and the higher speed will increase the risk of lethal ship strikes and noise.

The number of ships in the Arctic will influence the number of ship strikes too; more ships will result in more strikes. However, predicting the increase in ship traffic in the Arctic is difficult and predictions vary based on assumptions of future navigability and technical accessibility of the Arctic, the navigation season length, transit time, fuel consumption, the ability to adhere to schedules, and expansion within areas of oil, gas, and infrastructure development, which all relate to economic competitiveness of the new transit routes compared to traditional ones like the Suez and Panama canals or exploitation of the area [63]. While shipping activity has been increasing in the Arctic Ocean, most shipping currently revolves around providing services to local communities and few long-distance means of transport along the Northern Sea Route [46, 64, 65]. Azzara et al. [12] predict a 5-fold increase in U.S. maritime traffic in the Arctic between 2015 and 2025. Likewise, the Ocean Conservancy [63] state that

growth in vessel traffic can vary between 10% to 500%, which relates to low-growth and high-growth economic scenarios. Here, we assume that shipping will increase 5-fold in the next 20 years.

S4.2. Impacts of Shipping – Noise

The Arctic Ocean has thus far been less polluted by anthropogenic noise than other marine habitats around the world. The lack of ships and industrial development as a result of sea ice, as well as the influence of sea ice on noise propagation in the water, means the Arctic Ocean is a quieter acoustic refuge compared to industrialized waters [66-68]. The impacts of anthropogenic noise on marine organisms are diverse. Noise can mask or change the frequency of long-distance communication between conspecifics, change navigation routes, reduce the success of prey capture, result in hearing loss, increase energy expenditure through avoidance responses and increased travel times, result in temporary or permanent displacement with consequences for the use of resting places, reduce subsistence harvest success for indigenous communities, and increase animal stress levels or even result in death [8, 11, 44, 69-71]. Not all sound has an equal impact: chronic noise, especially low chronic noise from vessels, may have a greater influence on cetaceans in the long term than acute impulse noise from seismic surveys and pipe driving from the oil and gas industry, although those sounds can be lethal by causing brain lesions in organisms close by [71]. While many sources (such as exploration and extraction activities, construction and demolition activities, military activities and transportation) can increase noise, shipping is the source of the most widespread and continuous noise [53, 72]. Ships can affect a wide area with their noise, although the effect of the noise on an organism depends on the distance from the ship, the ship type, its speed, and the number of vessels present in the area [71, 73]. As a result, even large parts of a marine protected area may be impacted by noise when a shipping route is located nearby [73].

Whales endemic to the Arctic Ocean are sensitive to noise, having adapted to an environment with a unique soundscape. They may be particularly vulnerable to increased noise pollution from

anthropogenic activities in their environment [53]. Specific threshold noise levels have been determined that would result in behavioural changes when exceeded. These behavioural changes can be either temporary or permanent, depending on the level of the noise. NOAA [74] set the behavioural threshold for all marine mammals around the world at 120 dB, but Southall et al. [71] determined that bowhead whales show behavioural changes at noise levels of only 100 dB and severe behavioural changes at 140 dB. Specific data on noise thresholds for Arctic seals are limited, but Southall et al. [71] determined that 140 dB would likely result in behavioural changes in seals. The response of animals to noise will be influenced by the perceived level of noise, which is dependent on the weather. Windy days mask the noise of ships [73]. Furthermore, habituation to noise and the health of the animal may influence the response of the animal to the noise [75, 76]. These factors make it more difficult to determine the effect that noise can have on populations.

S4.3. Interaction Between Ship Strikes and Shipping Noise

The increase in shipping will influence both the risk of a strike and the effects of shipping noise, as described above. These two stressors, both combined in shipping activity, could be treated as independent effects. However, it is likely that the effects of both of these forcings interact. Whether the interactive effects will be antagonistic (cancel each other) or synergistic (amplify each other) is unknown. The two scenarios described below highlight why either of these impacts is possible (W. Halliday, personal communication). First, the noise of a ship may result in increased diving responses or displacement in whales, reducing the chance of a ship strike [53]. As such, shipping noise would lower the impact of ship strikes, resulting in an antagonistic interaction. This is likely to happen when bowhead whales are migrating (W. Halliday, personal communication). Alternatively, whales may tolerate and persist in a highly stressed state while the ships are present [14], becoming habituated to shipping noise and not moving away from the ship [53], resulting in a synergistic interaction. Bowhead whales in their feeding grounds may be unlikely to show avoidance responses and thus may be particularly

vulnerable to ship strikes (W. Halliday, personal communication). However, it is also thought that bowhead whales are very sensitive to noise and thus may not show habituation [53]. Clearly, the direction and magnitude of the interaction between shipping noise and ship strikes are unknown. Because OSIRIS is an exploratory tool and computationally light, we have elected to run a suite of different scenarios ranging from antagonistic to synergistic to see how the model responds to both kinds of interaction.

S5. Model validation

Given the paucity of population data for the Chukchi Sea, and the fact that we are forecasting 20 years into the future, validation of the OSIRIS model is difficult. However, it is still possible to compare modelled seasonal cycles and interannual trends produced for some nodes by the OSIRIS model to corresponding data collected in situ and from satellite remote sensing data. Here we show comparisons between model and in situ data for two lower trophic level nodes (nutrients and phytoplankton), both over an annual cycle and over a period of approximately two decades, and two high trophic level nodes (polar bears and walrus) over several decades (Supplemental Figure 7). In all cases, the agreement between the observations and results of the OSIRIS model is very good.

The annual cycle of nutrients produced by OSIRIS (assumed to be nitrate) is very similar to the pattern observed in surface waters of the Chukchi Sea [133-134]. Concentrations of nitrate remain high throughout the winter and drop rapidly in the spring as the phytoplankton bloom begins (Supplementary Figure 7A). Nitrate reaches very low concentrations by the peak of the phytoplankton bloom but increases rapidly in the autumn as wind-induced vertical mixing brings deep nutrients to the surface. Surface concentrations of phytoplankton measured from MODIS satellite data and produced by the OSIRIS model both exhibit the opposite trend to that of nitrate, showing very low concentrations in the winter and rapidly increasing in the spring, eventually peaking in the summer (Supplementary Figure 7C). Phytoplankton begin to drop as nitrate is depleted from surface waters, eventually reaching background levels in late autumn when

incident light levels fall to levels too low for net phytoplankton growth. In addition to producing an accurate annual cycle for phytoplankton, the OSIRIS model was also able to capture their long term trend. Over the 20-year simulations, the OSIRIS model forecasted a 30-40% increase in phytoplankton (Supplementary Figure 2), a value consistent with the 42% increase in phytoplankton productivity estimated for the Chukchi Sea between 1998 and 2012 [41].

To assess the behaviour of some of the upper trophic levels in the OSIRIS model, we looked at recent population trajectories for polar bear (Supplementary Figure 7B) and Pacific walrus Supplementary Figure 7D) and compared them to those forecasted by OSIRIS over the next 20 years. Population data suggest that polar bear abundance in the Beaufort Sea declined almost 2% per year [135], which would amount to a ~35% drop over 20 years. In contrast, populations of polar bear in the nearby Chukchi and Bering seas oscillated but remained virtually unchanged between the early 1970s and mid-2000s [136, 137]. This range in the estimated 20-year decline [0-35%] is consistent with the 10% decline in the polar bear numbers over 20 years forecasted by OSIRIS (Supplementary Figure 2). Similarly, populations of Pacific walrus have reportedly declined 0.9% per year or 18% per 20-year period (Supplementary Figure 7D) in the Chukchi and Bering seas [138-140]. This is on the same order as the 10% decline in Pacific walrus numbers forecast by OSIRIS over the next 20 years.

Supplementary Table 1. Daily intrinsic growth rates and variation (σ) per node used in the modelling scenarios.

Node name	Daily intrinsic growth rate	σ	References
Ice algae	0.1000	0.015	77
Picoplankton	0.0700	0.021	77
Diatoms	0.3000	0.015	77
Small zooplankton	0.0200	0.006	77
Large zooplankton	0.0300	0.009	77
Seabirds	0.0014	0.003	78, 79
Seals	0.0003	0.002	80
Polar bears	0.0002	0.000	80
Pacific salmon	0.0008	0.005	81-83
Arctic cod	0.0014	0.008	84
Bowhead whales	0.0001	0.001	80
Walrus	0.0002	0.001	80
Grey whales	0.0001	0.000	80
Amphipods	0.0205	0.006	85-87
Clams	0.0030	0.001	88
Nutrients	0.0200	0.006	77
Detritus	0.0800	0.002	77

Standard deviation (σ) indicates the uncertainty around the parameter values.

Supplementary Table 2. Node-node interaction coefficients used in the model.

Predatory nodes	Prey nodes	Value of predator effect on prey	σ	Value of prey effect on predator	σ	References & Notes
Ice algae	Nutrients	0.00500	0.00700	-0.00025	0.00000	77
Picoplankton	Nutrients	0.01000	0.01300	-0.10000	0.13000	77
Diatoms	Nutrients	0.10000	0.13000	-0.10000	0.13000	77
Small zooplankton	Ice algae	0.00620	0.00800	-0.00104	0.00100	77
Small zooplankton	Picoplankton	0.00600	0.00800	-0.00100	0.00100	77
Small zooplankton	Diatoms	0.00600	0.00800	-0.00450	0.00600	77
Large zooplankton	Ice algae	0.00016	0.00000	-0.00100	0.00130	77
Large zooplankton	Diatoms	0.00100	0.00100	-0.00750	0.01000	77
Large zooplankton	Small zooplankton	0.01650	0.02100	-0.02625	0.03400	77
Seabirds	Large zooplankton	0.00031	0.00300	-0.00440	0.04400	89-91
Seabirds	Arctic cod	0.00064	0.00600	-0.00036	0.00400	89-92
Seals	Arctic cod	0.00033	0.00300	-0.00058	0.00600	92-94
Polar bears	Seals	0.00012	0.00100	-0.00033	0.00300	95
Polar bears	Bowhead whales	0.00002	0.00000	-0.00011	0.00100	95
Polar bears	Walrus	0.00001	0.00000	-0.00022	0.00200	96
Polar bears	Grey whales	0.00001	0.00000	-0.00011	0.00100	95
Pacific salmon	Large zooplankton	0.00017	0.00200	-0.00500	0.05000	97
Pacific salmon	Arctic cod	0.00060	0.00600	-0.00042	0.00400	97
Arctic cod	Small zooplankton	0.00014	0.00100	-0.02000	0.20000	98
Arctic cod	Large zooplankton	0.00123	0.01200	-0.00950	0.09500	98
Bowhead whales	Small zooplankton	0.00003	0.00000	-0.01000	0.10000	99
Bowhead whales	Large zooplankton	0.00008	0.00100	-0.00810	0.08100	99
Bowhead whales	Arctic cod	0.00000	0.00000	-0.00001	0.00000	99
Bowhead whales	Amphipods	0.00000	0.00000	-0.00097	0.01000	99
Walrus	Clams	0.00022	0.00200	-0.00300	0.03000	100, 101
Grey whales	Large zooplankton	0.00004	0.00000	-0.00380	0.03800	102, 103
Grey whales	Amphipods	0.00007	0.00100	-0.01960	0.19600	102, 103
Amphipods	Detritus	0.02055	0.20600	-0.00030	0.00300	104-106
Clams	Detritus	0.00301	0.03000	-0.00100	0.00100	107-109
Diatoms	Ice algae	-0.01000	0.01300			
Detritus	Ice algae	0.00050	0.00500			
Detritus	Picoplankton	0.00010	0.00100			
Detritus	Diatoms	0.00050	0.00500			
Detritus	Small zooplankton	0.00050	0.00500			
Detritus	Large zooplankton	0.00050	0.00500			
Nutrients	Detritus	0.00030	0.00000			

Variation (σ) indicates the uncertainty around the parameter values. Several combinations do not represent predator-prey interactions: the influence of ice algae on diatom represents the competition between these two phytoplankton groups, rather than predator-prey interactions. The influence of phytoplankton and zooplankton groups and nutrients on detritus represents the contribution of these groups to the detritus pool.

Supplementary Table 3. Parameter values (a, b, or c) used to determine node responses to different forcings.

Node	Stressor														References
	Light				SST[1]						pH				
	a	σa	b	σb	a	σa	b	σb	c	σc	a	σa	b	σb	
Ice algae	4.000	4.320	-0.200	0.220	0.030	0.150	1.000	1.080			0.132	0.140	8.100	8.510	110, 111
Picoplankton	1.000	1.010	-0.100	0.110	0.070	0.080					0.132	0.140	8.100	8.510	110, 112
Diatoms	4.000	4.320	-0.100	0.110	0.070	0.080					0.132	0.140	8.100	8.510	110, 112
Small zooplankton					1.000	1.080	7.222	4.020	8.000	8.640	0.128	0.140	8.100	8.510	113
Large zooplankton					1.000	1.080	7.222	7.800	8.647	9.340	0.128	0.140	8.100	8.510	113
Seabirds															114-119
Seals															80, 115
Polar bears															80, 115, 120, 121
Pacific salmon					1.000	5.000	6.900	7.452	13.330	14.400	0.433	2.170	8.100	8.510	115, 122
Arctic cod					1.000	1.080	2.000	10.000	5.000	5.400	0.400	2.000	8.100	8.510	115, 123-125
Bowhead whales															80, 115, 126, 127
Walrus															80, 115, 127
Grey whales															80, 115, 126, 127
Amphipods					1.000	5.000	3.222	3.480	8.647	9.339	0.317	1.590	8.100	8.510	128
Clams					1.000	5.000	2.000	2.140	8.000	8.640	1.068	5.340	8.100	8.510	129

Node	Stressor												References
	Shipping noise		Ship strikes		OWD[2]				'Harvest'[3]		Inflow		
	a	σa	a	σa	a	σa	b	σb	a	σa	a	σa	
Ice algae													110, 111
Picoplankton											1.000	1.080	110, 112
Diatoms											0.500	0.540	110, 112
Small zooplankton											1.000	1.080	113
Large zooplankton											1.000	1.080	113
Seabirds	-0.015	0.080	-0.015	0.080									114-119
Seals	-0.004	0.020	-0.004	0.020	-0.002	0.010	1.287	1.390	-0.038	0.190			80, 115
Polar bears	-0.025	0.130			-0.001	0.010	1.143	1.235	-0.015	0.080			80, 115, 120, 121
Pacific salmon	-0.015	0.080							-0.050	0.250			115, 122
Arctic cod	-0.020	0.100			-0.003	0.010	1.430	1.544	-0.050	0.250			115, 123-125
Bowhead whales	-0.016	0.080	-0.016	0.080	-0.001	0.010	1.143	1.235	-0.019	0.100			80, 115, 126, 127
Walrus	-0.008	0.040	-0.008	0.040	-0.002	0.010	1.287	1.390	-0.020	0.100			80, 115, 127
Grey whales	-0.016	0.080	-0.016	0.080	-0.001	0.010	1.143	1.235	-0.031	0.160			80, 115, 126, 127
Amphipods													128
Clams													129

Standard deviation (σ) indicates the uncertainty around the parameter values. All forcing except responses to sea surface temperature (SST) are linear; SST responses follow a Gaussian functional response. The linear relationship of pH differs in its representation of the intercept. [1]SST = Sea surface temperature, [2]OWD = Open water duration, [3]'Harvest' = estimated from the biological removal potential, and is not limited to legally harvested potential.

Supplementary Table 4. Multiplicative values (interactions) per node and per pairwise stressor combination.

Node	Interaction	Model baseline	Model range	Literature range	References
Ice algae	SST[1] and pH	0.00	0.000	-3.072 to 0.704	110, 111
Ice algae	SST and Light	0.00	0.000	-3.103 to 0.248	111
Picoplankton	SST and pH	0.00	0.000	-3.072 to 0.700	110-112
Diatoms	SST and pH	0.00	0.000	-3.072 to 0.700	110-113
Small zooplankton	SST and pH	-0.30	-2.000 to 0.600	0.832 to 0.136	113
Small zooplankton	SST and Inflow	0.10	-0.050 to 0.050		
Small zooplankton	pH and Inflow	0.10	-0.050 to 0.050		
Large zooplankton	SST and pH	-0.30	-2.000 to 0.600	0.832 to 0.136	113
Large zooplankton	SST and Inflow	0.10	-0.050 to 0.050		
Large zooplankton	pH and Inflow	0.10	-0.050 to 0.050		
Seabirds	Ship strikes and Shipping noise	0.02	-0.050 to 0.050		
Seals	Ship strikes and Shipping noise	0.08	-0.500 to 0.500		80, 115, 127
Seals	Shipping noise and OWD[2]	0.05	-0.050 to 0.100		115, 127
Seals	'Harvest' and OWD	0.02	-0.050 to 0.050		80, 115
Polar bears	'Harvest' and OWD	0.02	0.000 to 0.050		80, 115, 120, 121
Polar bears	Shipping noise and OWD	0.02	-0.050 to 0.100		
Salmon	SST and pH	-0.10	-0.650 to 0.750	-0.650 to 0.750	122
Salmon	SST and 'Harvest'[3]	0.01	-0.100 to 0.100		
Arctic cod	SST and pH	-0.10	-0.650 to 1.000	-0.650 to 1.000	124
Arctic cod	SST and OWD	0.05	-0.050 to 0.200		115, 123, 125
Arctic cod	Noise and OWD	0.05	-0.050 to 0.200		115, 125
Arctic cod	'Harvest' and OWD	0.02	-0.100 to 0.100		115, 126
Bowhead whales	Ship strikes and Shipping noise	0.08	-0.500 to 0.500		80, 115, 126, 127
Bowhead whales	Shipping noise and OWD	0.05	-0.050 to 0.100		80, 127
Bowhead whales	'Harvest' and OWD	0.02	-0.050 to 0.050		80
Walrus	Ship strikes and Shipping noise	0.08	-0.500 to 0.500		80, 115, 127
Walrus	Shipping noise and OWD	0.05	-0.050 to 0.100		80, 115, 128
Walrus	'Harvest' and OWD	0.02	-0.050 to 0.050		80, 115
Grey whales	Ship strikes and Shipping noise	0.08	-0.500 to 0.500		80, 115, 126, 127
Grey whales	Shipping noise and OWD	0.05	-0.050 to 0.100		80, 115, 127
Grey whales	'Harvest' and OWD	0.02	-0.050 to 0.050		80
Amphipod	SST and pH	-0.50	-1.000 to 0.600	-1.937 to 0.565	128, 129
Clams	SST and pH	-0.20	-0.900 to 0.600	-0.938 to 0.584	130, 131

Negative values represent antagonistic values while positive values represent synergistic values. Model baseline refers to the baseline values indicated in the main paper, and the model range details the lower and upper bounds of the interaction values tested. Literature searches informed the interaction ranges where possible. In other cases, especially for marine mammals as well as the effect of noise, where interactions are expected to be present based on peer-reviewed studies investigating qualitative risks of one or both stressors, a range has been included to explore the potential effects of interactions between stressors. [1]SST = Sea surface temperature, [2]OWD = Open water duration, [3]'Harvest' = estimated from the biological removal potential, and is not limited to legally harvested potential.

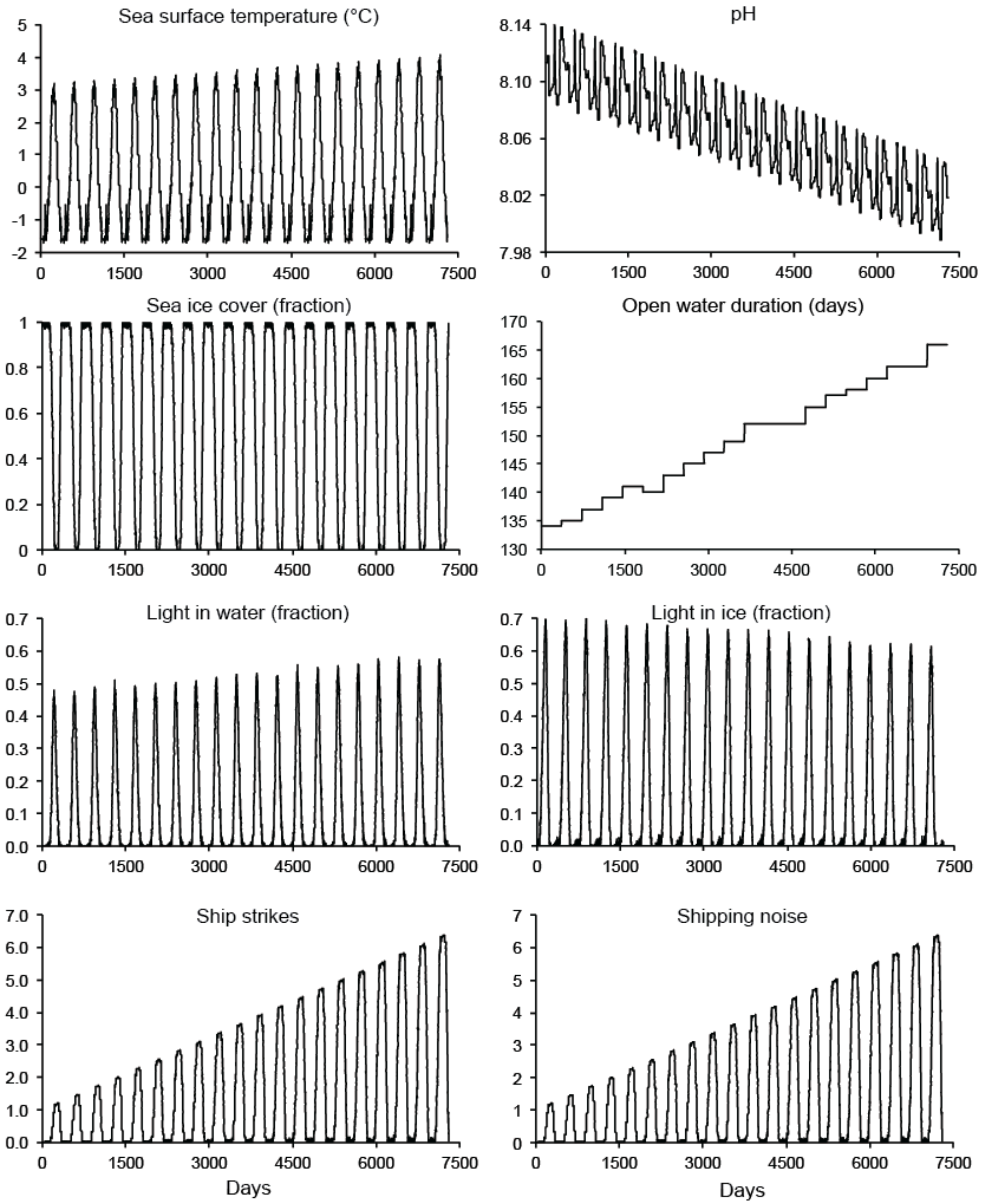

Supplementary Figure 1. The model forcing used during the 20-year simulations. See main text for details.

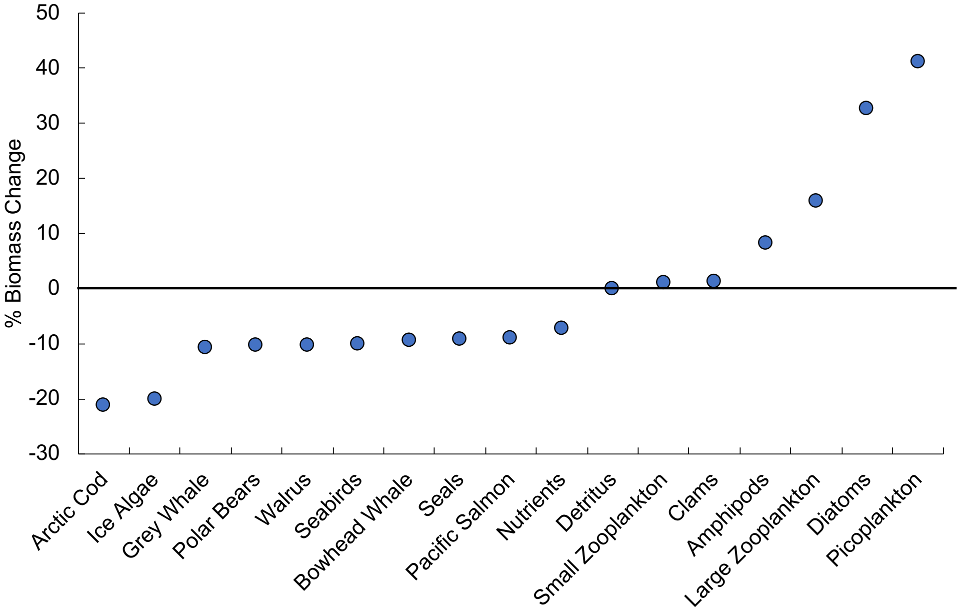

Supplementary Figure 2. The percent change in biomass for each node by the end of the 20-year baseline simulation.

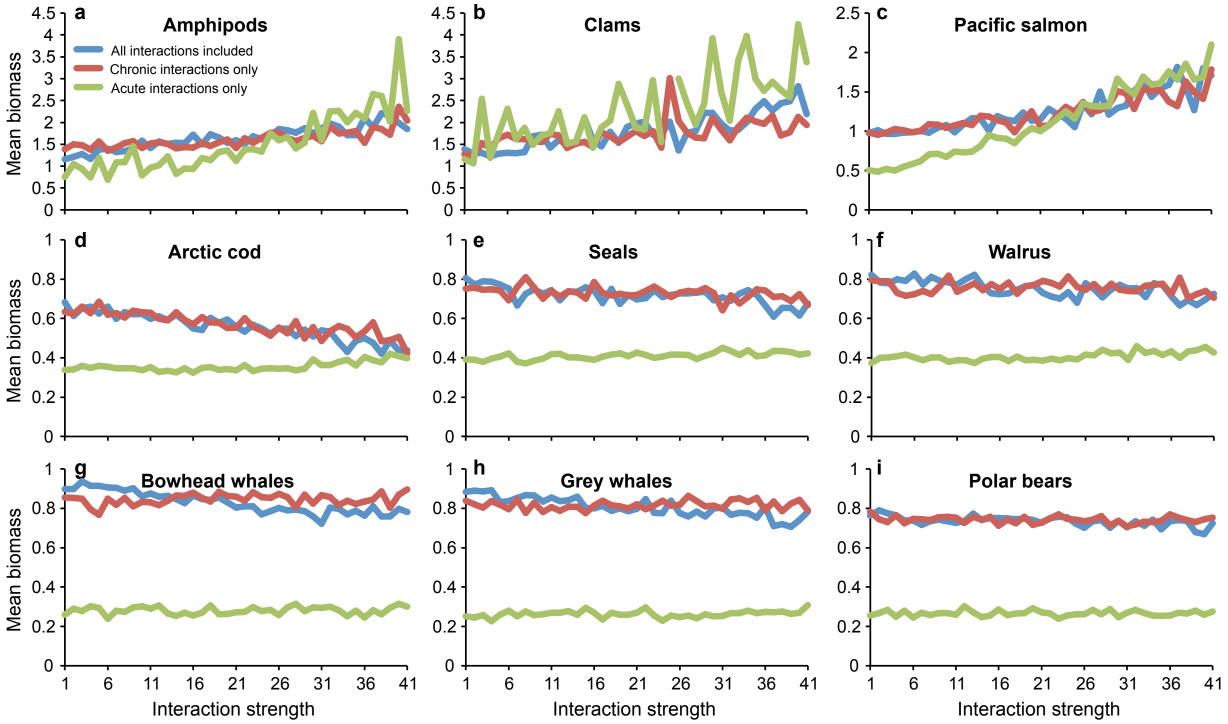

Supplementary Figure 3. Mean population size at the end of each 20-year simulation. Shown are data for the higher trophic level nodes averaged over the 200 runs performed at each of the 41 interaction strengths. A separate set of simulations was run with only acute stressor interactions included, only chronic stressor interactions included, and all stressor interactions included.

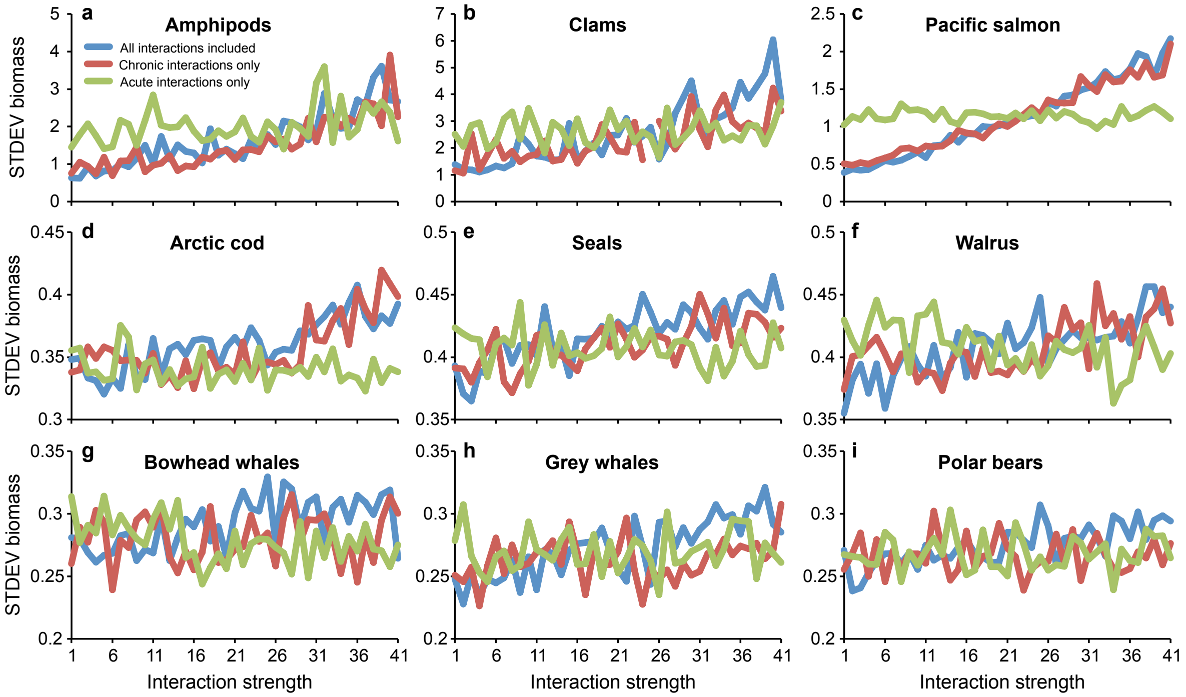

Supplementary Figure 4. Standard deviation of population size at the end of each 20-year simulation. Shown are data for the higher trophic level nodes calculated over the 200 runs performed at each of the 41 interaction strengths. A separate set of simulations was run with only acute stressor interactions included, only chronic stressor interactions included, and all stressor interactions included.

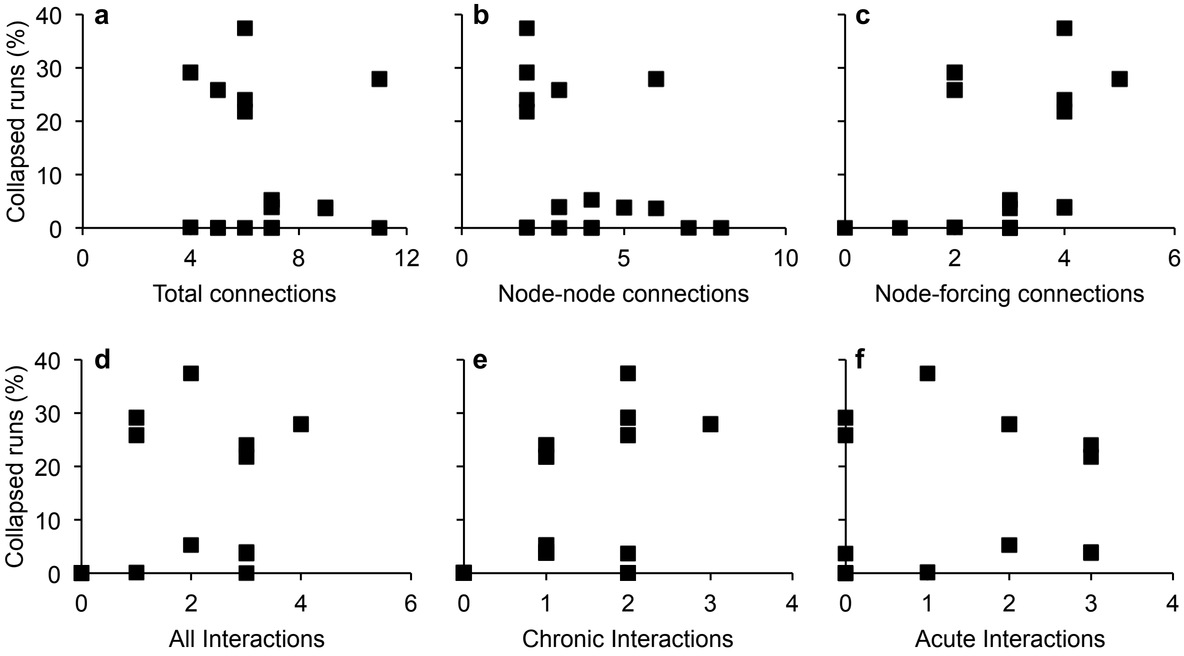

Supplementary Figure 5. The probability of population collapse. This is plotted as a function of the number of connections and interactions between nodes. Each point is calculated as the percent of 200 simulations where biomass of a node dropped below 10% of the starting value.

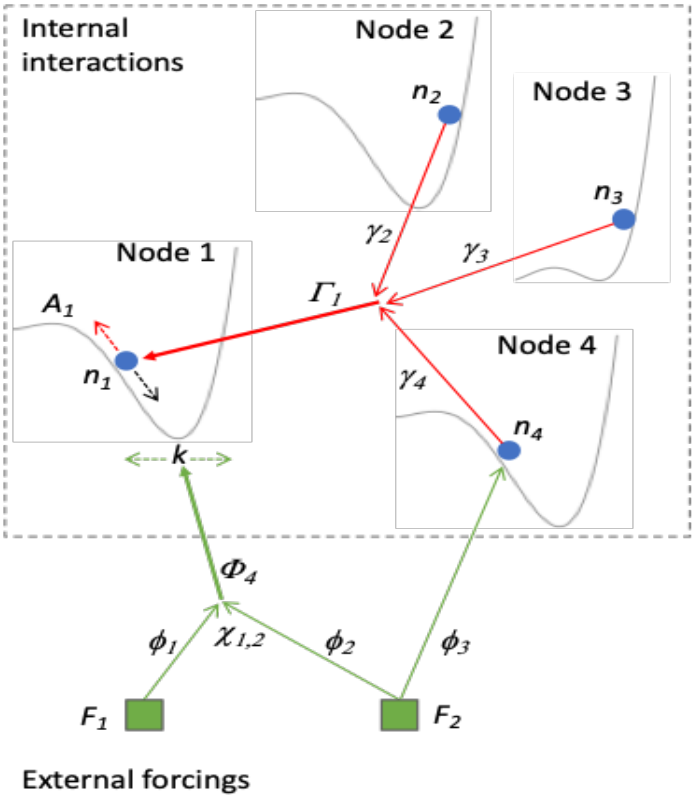

Supplementary Figure 6. A schematic representation of node equilibria and influences on nodes from [1]. Node states, n_i (blue circles), are determined by interactions with other nodes (internal interactions) and external forcing. When out of equilibrium, a node may exert an influence on another node (red arrows, γ_i, which combine to Γ_i). The dashed red arrow for Node 1 represents the influence the three other nodes have on this node, while the opposite black arrow represents the attraction to the equilibrium, k. External forcings may affect k, as indicated here by the individual forcing influences, ϕ_i, which may interact via χ. Note that unstable points, like A_1 for node 1, can be included. A represents an Allee effect.

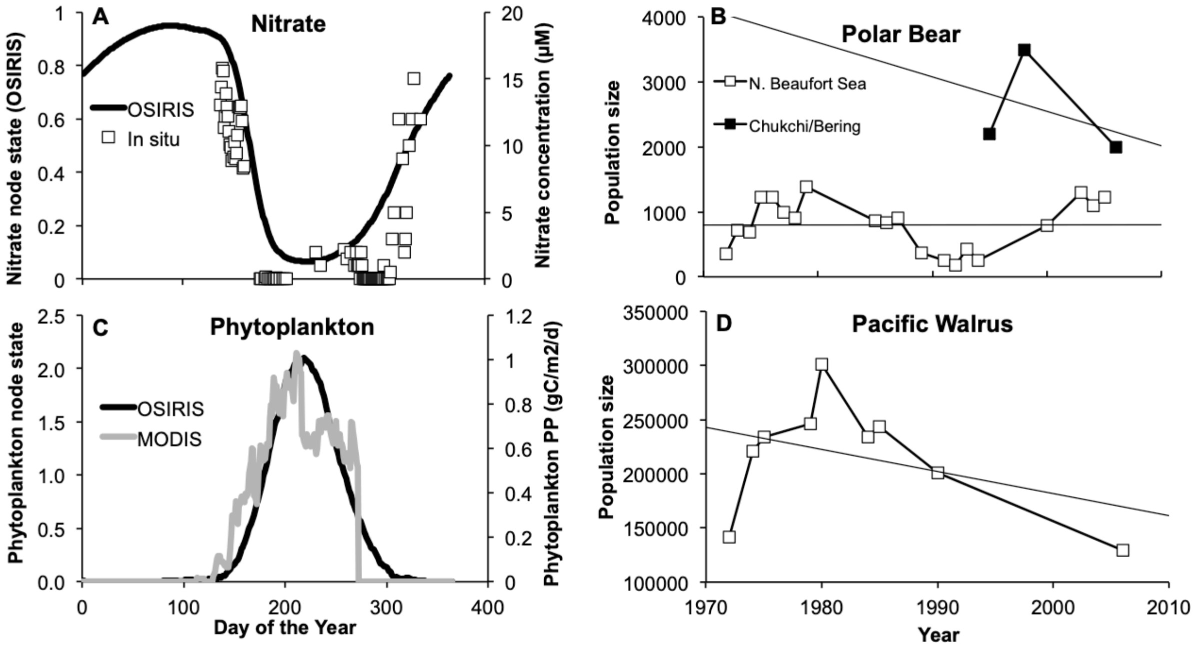

Supplemental Figure 7. Model validation. Comparison of the seasonal cycles of (A) nitrate and (C) phytoplankton produced by the baseline run of the OSIRIS model and measured in situ [132-134] and by satellite (MODIS). Also shown are the interannual changes in (B) polar bear [135-137] and (D) Pacific walrus populations in the vicinity of the Chukchi Sea [138-140] over the last several decades.

References

[1] Bailey, R. M. & van der Grient, J. M. A. A model for integrating the effects of multiple stressors on marine ecosystems. J Theor Biol **493**: https://doi.org/10.1016/j.jtbi.2020.110211 (2020).

[2] May, R.M., Will a large complex system be stable? Nature **238** (5364): 413–414. doi: 10.1038/238413a0 (1972).

[3] Feng, W. & Bailey, R.M. Unifying relationships between complexity and stability in mutualistic ecological communities. J Theor Biol 439: 100-126 (2018).

[4] Pörtner H-O. Oxygen- and capacity-limitation of thermal tolerance: a matrix for integrating climate-related stressor effects in marine ecosystems. J Exp Biol 213: 881-893 (2009).

[5] Busch, D. S., & McElhany P. Estimates of the direct effect of seawater pH on the survival rate of species groups in the California Current Ecosystem. PLoS ONE: 11: e0160669 (2016).

[6] Mathis, J. T. et al. Ocean acidification risk assessment for Alaska's fishery sector. Prog Oceanogr **136**: 71-91 (2015).

[7] Barnhart, K R., Miller, C. R., Overeem, I. & Kay, J. E. Mapping the future expansion of Arctic open water. Nat Clim Change **6**: 280-285 (2016).

[8] Reeves, R., Rosa, C., George, J. C., Sheffield, G. & Moore, M. Implications of Arctic industrial growth and strategies to mitigate future vessel and fishing gear impacts on bowhead whales. Mar Policy **36**: 454-462 (2012).

[9] George, J. C. et al. Frequency of injuries from line entanglements, killer whales, and ship strikes on Bering-Chukchi-Beaufort Seas bowhead whales. Arctic **70**: 37-46 (2017).

[10] Vanderlaan, A. S. M. & Taggart, C. T. Vessel collisions with whales: the probability of lethal injury based on vessel speed. Mar Mammal Sci **23**: 144-156 (2007).

[11] Lack, D. A. & Corbett, J. J. Black carbon from ships: a review of the effects of ship speed, fuel quality and exhaust gas scrubbing. Atmos Chem Phys **12**: 3985-4000 (2012).

[12] Azzara, A. J., Wang, H. & Rutherford, D. A 10-year projection of maritime activity in the U.S. Arctic region. The International Council on Clean Transportation. Washington, USA. 73 p (2015).

[13] Rolland, R. M. et al. Evidence that ship noise increases stress in right whales. Proc Biol Sci **279**: 2363-2368 (2012).

[14] Muto, M. M. et al. Alaska Marine Mammal Stock Assessments. U.S. Department of Commerce, NOAA Technical Memorandum NMFS-AFSC-378: 382p (2017).

[15] Côte, I. M., Darling, E. S. & Brown, C. J. Interactions among ecosystem stressors and their importance in conservation. P Roy Soc B-Biol Sci **283**: 20152592 (2016).

[16] Crain, C. M., Kroeker, K. & Halpern, B. S. Interactive and cumulative effects of multiple human stressors in marine systems. Ecol Lett **11**: 1304-1315 (2008).

[17] Darling, E. S. & Côté, I. M. Quantifying the evidence for ecological synergies**. Ecol Lett 11**: 1278–1286 (2008).

[18] Harvey, B. P., Gwynn-Jones, D & Moore, P. J. Meta-analysis reveals complex marine biological responses to the interactive effects of ocean acidification and warming. Ecol Evol **3**: 1016-1030 (2013).

[19] Kroeker, K. J. et al. Impacts of ocean acidification on marine organisms: quantifying sensitivities and interaction with warming. Glob Change Biol **19**: 1884-1896 (2013).

[20] Pigott, J. J., Townsend, C. R. & Matthaei, C. D. Reconceptualising synergism and antagonism among multiple stressors. Ecol Evol **5**: 1538-1547 (2015).

[21] Przeslawski, R., Byrne, M. & Mellin, C. A review and meta-analysis of the effects of multiple abiotic stressors on marine embryos and larvae. Glob Change Biol **21**: 2122-2140 (2015).

[22] Kunz, K. L. et al. New encounters in Arctic waters: a comparison of metabolism and performance of polar cod (*Boreogadus saida*) and Atlantic cod (*Gadus morhua*) under ocean acidification and warming. Polar Biol **39**: 1137-1153 (2016).

[23] Pongolini, L. et al. Assessing and Mitigating the Environmental Impacts of Shipping in the Arctic. Project Report http://commons.wmu.se/arctic_shipping/ (2017).

[24] Gradinger, R. & Bluhm, B. In-situ observations on the distribution and behaviour of amphipods and Arctic cod (*Boreogadus saida*) under the sea ice of the High Arctic Canada Basin. Polar Biol **27**: 595-603 (2004).

[25] Kovacs, K. M., Lydersen, C., Overland, J. E. & Moore, S. E. Impacts of changing sea-ice conditions on Arctic marine mammals. Mar Biodiv **41**: 181-194 (2011).

[26] Yurkowski, D., J. et al. Influence of sea ice phenology on the movement ecology of ringed seals across their latitudinal range. Mar Ecol Prog Ser **562**: 237-250 (2016).

[27] Post, E. Implications of earlier sea ice melt for phenological cascades in Arctic marine food webs. Food Webs **13**: 60-66 (2017).

[28] Stirling, I. & Smith, T. G. Implications of warm temperatures and an unusual rain event for the survival of ringed seals on the coast of Southeastern Baffin Island. Arctic **57**: 59-67 (2004).

[29] Bluhm, B. A. & Gradinger, R. Regional variability in food availability for arctic marine mammals. Ecol Appl **18**: S77-S96 (2008).

[30] Markus, T., Stroeve, J. C. & Miller J. Recent changes in Arctic sea ice melt onset, freezeup, and melt season length. J Geophys Res **114**: C12024 (2009).

[31] Stroeve, J. C., Markus, T., Boisvert, L., Miller, J. & Barrett, A. Changes in Arctic melt season and implications for sea ice loss. Geophys Res Lett **41**: 1216-1225 (2014).

[32] Douglas, D. C. Arctic sea ice decline: projected changes in timing and extent of sea ice in the Bering and Chukchi Seas. U. S. Geological Survey Open-File Report 2010-1176. 32 p (2010).

[33] Serreze, M. C., Crawford, A. D., Stroeve, J. C., Barrett, A. P. & Woodgate, R. A. Variability, trends and predictability of seasonal sea ice retreat and advance in the Chukchi Sea. J Geophys Res Oceans **121**: 7308-7325 (2016).

[34] Onarheim, I. H., Eldevik, T., Smedsrud, L. H. & Stroeve, J. C. Seasonal and regional manifestation of Arctic sea ice loss. J Clim **31**: 4917-4932 (2018).

[35] Maslanik, J., Stroeve, J., Fowler, C. & Emery, W. Distribution and trends in Arctic sea ice age through spring 2011. Geophys Res Lett **38**: L13502 (2011).

[36] Maslanik, J.A. et al. A younger, thinner Arctic ice cover: increased potential for rapid extensive sea ice loss. Geophys Res Lett **34**: 24501 (2007).

[37] ACIA. Arctic Climate Impact Assessment. Arctic Council and International Arctic Science Committee, Reykjavik (2004).

[38] Tremblay, J.-E., C., K. A., Gosselin, M. & Price, N. M. Bloom dynamics in early opening waters of the Arctic Ocean. Limnol Ocenaogr **51**: 900-912 (2006).

[39] Pabi, S., van Dijken, G. L. & Arrigo, K. R. Primary production in the Arctic Ocean, 1998-2006. J Geophys Res-Atmos **113**: C08005 (2008).

[40] Moline, M. A. et al. High latitude changes in ice dynamics and their impact on polar marine ecosystems. Ann NY Acad Sci **1134**: 267-319 (2008).

[41] Arrigo, K. R. & van Dijken, G. L. Continued increases in Arctic Ocean primary production. Prog Oceanogr **136**: 60-70 (2015).

[42] Arrigo, K. R. et al. Massive phytoplankton blooms under arctic sea ice. Science **336**: 1408 (2012).

[43] Søreide, J. E., Leu, E., Berge, J., Graeve, M. & Falk-Petersen, S. Timing of blooms, algal food quality and *Calanus glacialis* reproduction and growth in a changing Arctic. Glob Change Biol **16**: 3154-3163 (2010).

[44] Reeves, R., Rosa, C., George, J. C., Sheffield, G. & Moore, M. Implications of Arctic industrial growth and strategies to mitigate future vessel and fishing gear impacts on bowhead whales. Mar Policy **36**: 454-462 (2012).

[45] Reeves, R. R. et al. Distribution of endemic cetaceans in relation to hydrocarbon development and commercial shipping in a warming Arctic. Mar Policy **44**: 375-389 (2014).

[46] Pizzolato, L., Howell, S.E.L., Derksen, C., Dawson, J. & Copland, L. Changing sea ice conditions and marine transportation activity in Canadian Arctic waters between 1990 and 2012. Climate Change **123**: 161-173 (2014).

[47] Huntington, H. P. et al. Vessels, risks, and rules: planning for safe shipping in Bering Strait. Mar Policy **51**: 119-127 (2015).

[48] Kraus, S. D. Rates and potential causes of mortality in North Atlantic right whales (*Eubalaena glacialis*). Mar Mamm Sci **6**: 278-291 (1990).

[49] Knowlton, A. R. & Kraus, S. D. Mortality and serious injury of northern right whales (*Eubalaena glacialis*) in the western North Atlantic Ocean. J Cetacean Res Manag **2**: 193-208 (2001).

[50] Wiley, D. N., Asmutis, R. A., Pitchford, T. D. & Gannon, D. P. Stranding and mortality of humpback whales, *Megaptera novaeangliae*, in the mid-Atlantic and southeast United States, 1985-1992. Fish B-NOAA **93**: 196-205 (1995).

[51] Best, P. B., Peddemors, V. M., Cockcroft, V. G., & Rice, N. Mortalities of right whales and related anthropogenic factors in South Africa waters, 1963-1998. J Cetacean Res Manag **2**: 171-176 (2001).

[52] Moore, M. J., Knowlton, A. R., Kraus, S. D., McLellan, W. A. & Bonde, R. K. Morphometry, gross morphology and available histopathology in North Atlantic right whale (Eubalaena glacialis) mortalities (1970-2002). J Cetacean Res Manag **6**: 199–214 (2004).

[53] McWhinnie, L. H., Halliday, W. D., Insley, S. J., Hilliard, C. & Canessa, R. R. Vessel traffic in the Canadian Arctic: management solutions for minimizing impacts on whales in a changing northern region. Ocean Coast Manage **160**: 1-17 (2018).

[54] Vanderlaan, A. S. M. & Taggart, C. T. Vessel collisions with whales: the probability of lethal injury based on vessel speed. Mar Mammal Sci **23**: 144-156 (2007).

[55] Kraus, S. D. et al. Recent scientific publications cast doubt on North Atlantic right whale future. Front Mar Sci **3**: 137 (2016).

[56] Taylor, S. & Walker, T. R. North Atlantic right whales in danger. Science **358**: 730-731 (2017).

[57] Record, N. R. et al. Rapid climate-driven circulation changes threaten conservation of endangered North Atlantic right whales. Oceanography **32**: 162-169 (2019).

[58] Huntington, H. P. A preliminary assessment of threats to arctic marine mammals and their conservation in the coming decades. Mar Policy **33**: 77-82 (2009).

[59] Reimer, J., Gravel, C., Brown, M.W. & Taggart, C. T. Mitigating vessel strikes: the problem of peripatetic whales and the peripatetic fleet. Mar Policy **68**: 91–99 (2016).

[60] Conn, P. B. & Silber, G. K. Vessel speed restrictions reduce risk of collision-related mortality for North Atlantic right whales. Ecosphere **4**: 1-16 (2013).

[61] Bureau of Ocean Energy Management (BOEM). Chukchi Sea. Lease Sale 193. Final Second Supplemental Environmental Impact Statement. Volume 1. Chapters 1-7. 844 p. (2015).

[62] Pine, M. K., Hannay, D. E., Insley, S. J., Halliday, W. D. & Juanes, F. Assessing vessel slowdown for reducing auditory masking for marine mammals and fish of the western Canadian Arctic. Mar Pollut Bull **135**: 290-302 (2018).

[63] Ocean Conservancy. Navigating the North: an assessment of the environmental risks of Arctic vessel traffic. Anchorage, UK. 87 pp (2017).

[64] Pizzolato, L., Howell, S., Dawson, J., Laliberté, F. & Copland, L. The influence of declining sea ice on shipping activity in the Canadian Arctic. Geophys Res Lett **43**: 146-154 (2016).

[65] Zhang, Y., Meng, Q. & Zhang, L. Is Northern Sea Route attractive to shipping companies? Some insights from recent ship traffic data. Mar Policy **73**: 53-60 (2016).

[66] Roth, E. H., Hildebrand, J. A., Wiggins, S. M. & Ross, D. Underwater ambient noise on the Chukchi Sea continental slope from 2006-2009. J Acoust Soc Am **131**: 104-110 (2012).

[67] Kinda, G. B., Simard, Y., Gervaise, C., Mars, J. I. & Fortier, L. Under-ice ambient noise in Eastern Beaufort Sea, Canadian Arctic, and its relation to environmental forcing. J Acoust Soc Am **134**: 77-87 (2013).

[68] Insley, S. J., Halliday, W. D. & de Jong, T. Seasonal patterns in ocean ambient noise near Sachs Harbour, Northwest Territories. Arctic **70**: 239-248 (2017).

[69] Erbe, C. & Farmer, D. M. Zones of impact around icebreakers affecting beluga whales in the Beaufort Sea. J Acoust Soc Am **108**: 1332-1340 (2000).

[70] Findley, L. T. & Vidal, O. Gray whale (Eschrichtus robustus) at calving sites in the Gulf of California, Mexico. J Cetacean Res Manag **4**: 27–40 (2002).

[71] Southall, B. L. et al. Marine mammal noise criteria: initial scientific recommendations. Aquat Mamm **33**: 411-509 (2007).

[72] McDonald, M. A., Hildebrand, J.A. & Wiggins, S. M. Increases in deep ocean ambient noise in the Northeast Pacific west of San Nicolas Island, California. J Acoust Soc Am **120**: 711-718 (2006).

[73] Halliday, W. D., Insley, S. J., Hilliard, R. C., de Jong, T. & Pine, M. K. Potential impacts of shipping noise on marine mammals in the western Canadian Arctic. Mar Pollut Bull **123**: 73-82 (2017).

[74] National Oceanic and Atmospheric Administration. Interim sound threshold guidance. [WWW Document]. URL. http://www/westcoast.fisheries.noaa.gov/protected_species/marine_mammals/ threshold_guidance.html (2016)

[75] Ellison, W. T., Southall, B. L., Clark, C. W. & Frankel, A. S. A new context-based approach to assess marine mammal behaviour responses to anthropogenic sounds. Conserv Biol **26**: 21-28 (2012).

[76] Gomez, C. et al. A systematic review on the behavioural responses of wild marine mammals to noise: the disparity between science and policy. Can J Zool **819**: cjz-2016-0098 (2016).

[77] Palmer, M. A., Saenz, B. T. & Arrigo, K. R. Impacts of sea ice retreat, thinning, and melt-pond proliferation on the summer phytoplankton bloom in the Chukchi Sea, Arctic Ocean. Deep-sea Res **105**: 85-104 (2014).

[78] Divoky, G. J., Lukacs, P. M. & Druckenmiller, M. L. Effects of recent decreases in arctic sea ice on an ice-associated marine bird. Prog Oceanogr **136**: 151-161 (2015).

[79] Anderson, C. M., et al. Modelling demographic impacts of a growing Arctic fishery on a seabird population in Canada and Greenland. Mar Environ Res **142**: 80-90 (2018).

[80] Muto, M. M. et al. Alaska Marine Mammal Stock Assessments. U.S. Department of Commerce, NOAA Technical Memorandum NMFS-AFSC-378: 382p (2017).

[81] Gjedrem, T. & Gunnes, K. Comparison of growth rate in Atlantic salmon, pink salmon, Arctic char, sea trout and rainbow trout under Norwegian farming conditions. Aquaculture **13**: 135-141 (1978).

[82] Lewis, B., Grant, W. S., Brenner, R. E. & Hamazaki, T. Changes in size and age of Chinook salmon *Oncorhynchus tshawytscha* returning to Alaska. PLoS ONE **10**: e0130184 (2015).

[83] Ricker, W. E. Review of the rate of growth and mortality of Pacific salmon in salt water, and noncatch mortality caused by fishing. J of the Fish Board of Canada **33.7**: 1483-1524 (1976).

[84] Hop, H., Tonn, W. M. & Welch, H. E. Bioenergetics of Arctic cod (*Boreogadus saida*) at low temperatures. Canadian Journal of Fisheries and Aquaculture Sciences **54**: 1772-1784 (1997).

[85] Percy, J. A. Reproduction and growth of the Arctic hyperiid amphipod *Themisto libellula* Mandt. Polar Biol **13**: 131-139 (1993).

[86] Poltermann, M. Growth, production and productivity of the Arctic sympagic amphipod *Gammarus wilkitzkii*. Mar Ecol Prog Ser **193**: 109-116 (2000).

[87] Brown, K. E., King, C. K. & Harrison, P. L. Reproduction, growth and early life history of the Antarctic gammarid amphipod *Paramoera walker*. Polar Biol **38**: 583-1596 (2015).

[88] Sejr, M. K. et al. Growth and production of *Hiatella arctica* (Bivalvia) in a high-Arctic fjord (Young Sound, Northeast Greenland). Mar Ecol Prog Ser **244**: 163-169 (2002).

[89] Mehlum, F. & Gabrielsen, G. W. The diet of High-Arctic seabirds in coastal and ice-covered, pelagic areas near the Svalbard archipelago. Polar Res **12**: 1:20 (1993).

[90] Provencher, J. F., Gaston, A. J., O'Hara, P. D. & Gilchrist, H. G. Seabird diet indicates changing Arctic marine communities in eastern Canada. Mar Ecol Prog Ser **454**: 171:182 (2012).

[91] Dummond, B. A. Summary figures of diet data from birds on the Alaska Maritime National Wildlife Refuge. U.S. Fish and Wild. Serv. Rep., AMNWR 2016/06. Horner Alaska (2016).

[92] Lowry, L. F., Frost, K. J., Burns, J. J. Feeding of bearded seals in the Bering and Chukchi Seas and trophic interaction with Pacific walruses. Arctic **33**: 330:342 (1980).

[93] Finley, K. J. & Evans, C.R. Summer diet of the bearded seal (*Erignathus barbatus*) in the Canadian High Arctic. Arctic **36**: 82-89 (1983).

[94] Finley, K. J., Bradstreet, M. S. W. & Miller, G. W. Summer feeding ecology of harp seals (*Phoca groenlandica*) in relation to Arctic cod (*Boreogadus saida*) in the Canadian High Arctic. Polar Biol **10**: 609-618 (1990).

[95] Rode, K.D. et al. Variation in the response of an Arctic top predator experiencing habitat loss: feeding and reproductive ecology of two polar bear populations. Glob Change Biol **20**: 76-88 (2014).

[96] Calvert, W. & Stirling, I. Interactions between polar bears and overwintering walruses in the Central Canadian High Arctic. International Conference on bear research and management. In: Bears: their biology and management **8**: 351-356 (1990).

[97] Moss, J. H., Murphy, J. M., Farley Jr., E. V., Eisner, L. B. & Andrews, A.G. Juvenile pink and chum salmon distribution, diet and growth in the northern Bering and Chukchi Seas. N Pacific Anadromous Fish Commission Bull **5**: 191:196 (2009).

[98] Buckley, T. W. & Whitehouse, G. A. Variation in the diet of Arctic cod (*Boreogadus saida*) in the Pacific Arctic and Bering Sea. Environ Biol Fish **100**: 421:442 (2017).

[99] Lowry, L. F., Sheffield, G. & George, J. C. Bowhead whale feeding in the Alaskan Beaufort Sea, based on stomach contents analyses. J Cetacean Res Manage **63**: 215-223 (2004).

[100] Sheffield, G., Fay, F. H., Feder, H. & Kelly, B. P. Laboratory digestion of prey and interpretation of walrus stomach contents. Mar Mamm Sci **17**: 310-330 (2006).

[101] Dehn, L.-A. et al. Feeding ecology of phocid seals and some walrus in the Alaskan and Canadian Arctic as determined by stomach contents and stable isotope analysis. Polar Biol **30**: 167-181 (2007).

[102] Budnikova, L. L. & Blokhin, S. Food contents of the eastern grey whale *Eschrichtius robustus* Lilljeborh, 1861 in the Mechigmensky Bay of the Bering Sea. Russ J Mar Biol **38**: 149-155 (2012).

[103] Brower, A. A. et al. Gray whale distribution relative to benthic invertebrate biomass and abundance: Northeastern Chukchi Sea 2009-2012. Deep-Sea Res **144**: 156-174 (2017).

[104] Poltermann, M. Arctic sea ice as feeding ground for amphipods – food sources and strategies. Polar Biol **24**: 89-96 (2001).

[105] Sainte-Marie, B. Feeding and swimming of lysianassid amphipods in a shallow cold-water bay. Mar Biol **91**: 219-229 (1986).

[106] Nygård, H., Berge, J., Søreide, J. E., Vihtakari, M. & Falk-Petersen, S. The amphipod scavenging guild in two Arctic fjords: seasonal variations, abundances and trophic interactions. Aquat Biol **14**: 247-264 (2012).

[107] Carey Jr., A. G., Scott, P. H. & Walters, K. R. Distributional ecology of shallow southwestern Beaufort Sea (Arctic Ocean) bivalve Mollusca. Mar Ecol Prog Ser **17**: 125-134 (1984).

[108] Berge, J. et al. Unexpected levels of biological activity during the polar night offer new perspective on a warming Arctic. Curr Biol **25**: 2555-2561 (2015).

[109] Sjer, M. K., Petersen, J. K., Jensen, K. T. & Rysgaard, S. Effects of food concentration on clearance rate and energy budget of the Arctic bivalve *Hiatella arctica* (L) at subzero temperature. J Exp Mar Biol Ecol **311**: 171-183 (2004).

[110] Coello-Camba, A., Agusti, S., Holding, J., Arrieta, J. M. & Duarte, C. M. Interactive effect of temperature and CO_2 increase in Arctic phytoplankton. Front Mar Sci **1**: 1-10 (2014).

[111] Rajanahally, M. A., Sim, D., Ryan, K. G. & Convey, P. Can bottom ice algae tolerate irradiance and temperature changes? J Exp Mar Biol Ecol **461**: 516-527 (2014).

[112] Keys, M., Tilstone, G., Findlay, H. S., Widdicombe, C. E. & Lawson, T. Effects of elevated CO_2 and temperature on phytoplankton community biomass, species composition and photosynthesis during an experimentally induced autumn bloom in the western English Channel. Biogeosciences **15**: 3203-3222 (2018).

[113] Hildebrant, N., Niehoff, B. & Sartoris, J. Long-term effects of elevated CO_2 and temperature on the Arctic calanoid copepods *Calanus glacialis* and *C. hyperboreus*. Mar Pollut Bull **80**: 59-70 (2014).

[114] Gaston, A. J. et al. Modelling foraging range for breeding colonies of thick-billed murres *Uria lomvia* in the Eastern Canadian Arctic and potential overlap with industrial development. Biol Conserv **168**: 134-143 (2013).

[115] Bureau of Ocean Energy Management (BOEM). Chukchi Sea. Lease Sale 193. Final Second Supplemental Environmental Impact Statement. Volume 1. Chapters 1-7. 844p. (2015).

[116] Huntington, H. P. et al. Vessels, risks, and rules: planning for safe shipping in Bering Strait. Mar Policy **51**: 119-127 (2015).

[117] Renner, M. & Kuletz, K. J. A spatial-seasonal analysis of the oiling risk from shipping traffic to seabirds in the Aleutian Archipelago. Mar Pollut Bull **101**: 127-136 (2015).

[118] Andersen, J. H. et al. Potential for cumulative effects of human stressors on fish, sea birds and marine mammals in Arctic waters. Estuar Coast Shelf S **184**: 202-206 (2017).

[119] Wong, S. N. P., Gjerdum, C., Gilchrist, H. G. & Mallory, M. L. Seasonal vessel activity risk to seabirds in waters off Baffin Island, Canada. Ocean Cost Manage **163**: 339-351 (2018).

[120] Derocher, A. E., Lunn, N. J. & Stirling, I. Polar bears in a warming climate. Integr Comp Biol **442** 163-176 (2004).

[121] Durner, G. M., Douglas, D. C., Nielson, R. M., Amstrup, S. C. & McDonald, T. L. Predicting the future distribution of polar bear habitat in the Polar Basin from resource

selection functions applied to 21st Century General Circulation Model Projections of sea ice. U.S. Geological Survey, Reston, Virginia. 61 pp. (2007).

[122] Eisner, L., Hillgruber, N., Martinson, E., Maselko J. Pelagic fish and zooplankton species assemblages in relation to water mass characteristics in the north Bering and southeast Chukchi Seas. Polar Biol **36**: 87-113 (2013).

[123] Hop, H. & Gjøsæter, H. Polar cod (*Boreogadus saida*) and capelin (*Mallotus villosus*) as key species in marine food webs of the Arctic and the Barents Sea. Mar Biol Res **9**: 878-894 (2013).

[124] Kunz, K. L. et al. New encounters in Arctic waters: a comparison of metabolism and performance of polar cod (*Boreogadus saida*) and Atlantic cod (*Gadus morhua*) under ocean acidification and warming. Polar Biol **39**: 1137-1153 (2016).

[125] De Robertis, A., Taylor K., Wilson, C. D. & Farley, E. V. Abundance and distribution of Arctic cod (*Boreogadus saida*) and other pelagic fishes over the U.S. Continental Shelf of the Northern Bering and Chukchi Seas. Deep-Sea Res **135**: 51-65 (2017).

[126] Rolland, R. M. et al. Evidence that ship noise increases stress in right whales. Proc Biol Sci **279**: 2363-2368 (2012).

[127] Hauser D. D. W., Laidre, K. L. & Stern, H. L. Vulnerability of Arctic marine mammals to vessel traffic in the increasingly ice-free Northwest Passage and Northern Sea Route. PNAS **115**: 7617-7622 (2018).

[128] Cardoso, P. G. et al. Interactive effects of increased temperature, pCO_2 and the synthetic progestin levonorgestrel on the fitness and breeding of the amphipod *Gammarus locusta*. Environ Pollut **236**: 937-947 (2018).

[129] Schram, J. B., Schoenrock, K. M., McClintock, J. B., Amsler, C. D. & Angus R. A. Seawater acidification more than warming presents a challenge for two Antarctic macroalgal-associated amphipods. Mar Ecol Prog Ser **554**: 81-97 (2016).

[130] Talmage S. C. & Gobler C. J. Effects of past, present and future ocean carbon dioxide concentrations on the growth and survival of larval shellfish. PNAS **107**: 17246-17251 (2010).

[131] Talmage S. C. & Gobler C. J. Effects of elevated temperature and carbon dioxide on the growth and survival of larvae and juveniles of three species of northwest Atlantic bivalves. PLoS ONE **6**: E26941 (2011).

[132] Lowry, K. E., Pickart R. S., Mills M. M., Brown Z. W., van Dijken G. L., Bates N. R., & Arrigo K. R. The influence of winter water on phytoplankton blooms in the Chukchi Sea. Deep-Sea Research, Part II, **118**: 53-72, doi: 10.1016/j.dsr2.2015.06.006 (2015).

[133] Arrigo, K. R., Mills M. M., van Dijken G. L., Lowry K. E., Pickart R. S., & Schlitzer R. Late spring nitrate distributions beneath the ice-covered northeastern Chukchi Shelf. Journal of Geophysical Research: Biogeosciences, **122**: 2409-2417, doi:10.1002/2017JG003881 (2017).

[134] Mordy, C. W, Bell S., Cokelet E. D., Ladd C., Lebon G., Proctor P., Stabeno P., Strausz D., Wisegarver E., & Wood K. Seasonal and interannual variability of nitrate in the eastern Chukchi Sea: Transport and winter replenishment. DeepSea Res. Part II, doi:https://doi.org/10.1016/j.dsr2.2020.104807 (2020).

[135] Stirling, I., McDonald T. L., Richardson E. S., Regehr E. V., & Amstrup S. C. Polar bear population status in the northern Beaufort Sea. Ecol. Appl. 21: 859-876 (2011).

[136] U.S. Fish and Wildlife Service Marine Mammals Management. Polar Bear (*Ursus maritimus*): Chukchi/Bering Seas Stock. Stock Assessment Reports (2002).

[137] Allen B. M. & Angliss R. P. NOAA Polar Bear (*Ursus maritimus*): Chukchi/Bering Seas Stock. Alaska Marine Mammal Stock Assessments (2010).

[138] Garner, G. W. Pacific Walruses. In Our living resources: a report to the nation on the distribution, abundance and health of U.S. plants, animals, and ecosystems (Edited by LaRoe E. T., Farris G. T., Puckett C. E., Doran P.D., and Mac M. J.) U.S. Department of the Interior, National Biological Service, Washington, DC (1995).

[139] Fay, F. H., Eberhardt L. L., Kelly B. P., Burns J. J. & Quakenbush L. T. Status of the Pacific walrus population, 1950-1989. Mar. Mammal Sci. **13**(4): 537-565 (1997).

[140] Allen, B. M. & Angliss R. P. NOAA Pacific Walrus (*Odobenus rosmarus divergens*): Alaska Stock. Alaska Marine Mammal Stock Assessments (2010).
