# Supplementary material for: Synergistic interactions among growing stressors increase risk to an Arctic ecosystem
Source: Nat Commun. 2020 Dec 7;11:6255. doi: 10.1038/s41467-020-19899-z (PMC7721797; doi:10.1038/s41467-020-19899-z)
Supplement: Supplementary file 2 — Reporting Summary [file 41467_2020_19899_MOESM2_ESM.pdf]

nature research

Corresponding author(s): Kevin Arrigo
Last updated by author(s): Oct 23, 2020

# Reporting Summary

Nature Research wishes to improve the reproducibility of the work that we publish. This form provides structure for consistency and transparency in reporting. For further information on Nature Research policies, see Authors & Referees and the Editorial Policy Checklist .

## Statistics

For all statistical analyses, confirm that the following items are present in the figure legend, table legend, main text, or Methods section.

| n/a | Confirmed | |
|---|---|---|
| ☐ | ☒ | The exact sample size (*n*) for each experimental group/condition, given as a discrete number and unit of measurement |
| ☒ | ☐ | A statement on whether measurements were taken from distinct samples or whether the same sample was measured repeatedly |
| ☒ | ☐ | The statistical test(s) used AND whether they are one- or two-sided<br>*Only common tests should be described solely by name; describe more complex techniques in the Methods section.* |
| ☒ | ☐ | A description of all covariates tested |
| ☒ | ☐ | A description of any assumptions or corrections, such as tests of normality and adjustment for multiple comparisons |
| ☐ | ☒ | A full description of the statistical parameters including central tendency (e.g. means) or other basic estimates (e.g. regression coefficient) AND variation (e.g. standard deviation) or associated estimates of uncertainty (e.g. confidence intervals) |
| ☒ | ☐ | For null hypothesis testing, the test statistic (e.g. *F*, *t*, *r*) with confidence intervals, effect sizes, degrees of freedom and *P* value noted<br>*Give P values as exact values whenever suitable.* |
| ☒ | ☐ | For Bayesian analysis, information on the choice of priors and Markov chain Monte Carlo settings |
| ☒ | ☐ | For hierarchical and complex designs, identification of the appropriate level for tests and full reporting of outcomes |
| ☒ | ☐ | Estimates of effect sizes (e.g. Cohen's *d*, Pearson's *r*), indicating how they were calculated |

*Our web collection on statistics for biologists contains articles on many of the points above.*

## Software and code

Policy information about availability of computer code

| | |
|---|---|
| Data collection | We used the OSIRIS (Ocean System Interactions, Risks Instabilities, and Synergies) model to investigate the importance of synergistic interactions between stressors in the Arctic Ocean over a 20 year period. The model was developed by Bailey & Van der Grient, https://doi.org/10.1016/j.jtbi.2020.110211 . |
| Data analysis | Analyses of the results were done with IDL 8.7.0 (Harris Geospatial Solutions, Inc.) and Microsoft Excel (version 16.34 (20020900)). See also the nr-software-policy.pdf document. |

For manuscripts utilizing custom algorithms or software that are central to the research but not yet described in published literature, software must be made available to editors/reviewers. We strongly encourage code deposition in a community repository (e.g. GitHub). See the Nature Research guidelines for submitting code & software for further information.

## Data

Policy information about availability of data

All manuscripts must include a data availability statement. This statement should provide the following information, where applicable:

- Accession codes, unique identifiers, or web links for publicly available datasets
- A list of figures that have associated raw data
- A description of any restrictions on data availability

Model results presented in this paper and forcing data used to drive the OSIRIS model are available at the permanent Stanford Digital Depository (https://purl.stanford.edu/js210vj5991). The source data underlying Figures 2-4 and Supplementary Figures 1-5 are provided as a Source Data file.

# Field-specific reporting

Please select the one below that is the best fit for your research. If you are not sure, read the appropriate sections before making your selection.

☐ Life sciences ☐ Behavioural & social sciences ☒ Ecological, evolutionary & environmental sciences

For a reference copy of the document with all sections, see nature.com/documents/nr-reporting-summary-flat.pdf

# Ecological, evolutionary & environmental sciences study design

All studies must disclose on these points even when the disclosure is negative.

| | |
|---|---|
| Study description | We used a network interaction model to study stressor interactions explicitly. We focus on the Arctic marine environment due to the extensive climate-driven stress of sea ice loss that accelerates the growth of stressors from human industries, including shipping and oil exploration. |
| Research sample | We ran a series of 200 miltiple-stresor simulations at each of 41 different stressor interaction strengths. This was done three times with acute strassors only, chronic stressors only, and both chronic and acute stressors included. |
| Sampling strategy | This was a modeling study and no sampling was done. |
| Data collection | No data were collected as part of this study. |
| Timing and spatial scale | 20 year simulation of the central Chukchi Sea. |
| Data exclusions | No data were excluded |
| Reproducibility | Each simulation was run independently. |
| Randomization | We used Latin hypercube sampling to create the 200 simulations where we randomly varied the internal parameter set. |
| Blinding | This was a modeling study and blinding was not used. |

Did the study involve field work? ☐ Yes ☒ No

# Reporting for specific materials, systems and methods

We require information from authors about some types of materials, experimental systems and methods used in many studies. Here, indicate whether each material, system or method listed is relevant to your study. If you are not sure if a list item applies to your research, read the appropriate section before selecting a response.

## Materials & experimental systems

| n/a | Involved in the study |
|---|---|
| ☒ | ☐ Antibodies |
| ☒ | ☐ Eukaryotic cell lines |
| ☒ | ☐ Palaeontology |
| ☒ | ☐ Animals and other organisms |
| ☒ | ☐ Human research participants |
| ☒ | ☐ Clinical data |

## Methods

| n/a | Involved in the study |
|---|---|
| ☒ | ☐ ChIP-seq |
| ☒ | ☐ Flow cytometry |
| ☒ | ☐ MRI-based neuroimaging |
